# Structural Requirements of 1-(2-Pyridinyl)-5-pyrazolones for Disproportionation of Boronic Acids

**DOI:** 10.3390/molecules26226814

**Published:** 2021-11-11

**Authors:** Joungmo Cho, Venkata Subbaiah Sadu, Yohan Han, Yunsoo Bae, Hwajeong Lee, Kee-In Lee

**Affiliations:** 1 Korea Research Institute of Chemical Technology, Daejeon 34114, Korea; jmcho@krict.re.kr (J.C.); yhhan@krict.re.kr (Y.H.); 2 R&D Center, Molecules & Materials Co., Ltd., B-219 Daeduck BIZ Center, Daejeon 34013, Korea; subboo32@outlook.com; 3 Department of Life Science, Ewha Womans University, Seoul 03760, Korea; baeys@ewha.ac.kr; 4 Graduate School of Pharmaceutical Sciences, Ewha Womans University, Seoul 03760, Korea; hwalee@ewha.ac.kr

**Keywords:** 1-(2-pyridinyl)-5-pyrazolone, [*N*,*O*]-bidentate ligand, arylboronic acid, base, diarylborination, four-coordinate boron(III) complex

## Abstract

We observed an unusual formation of four-coordinate boron(III) complexes from the reaction of 1-(2-pyridinyl)-5-pyrazolone derivatives with arylboronic acids in the basic media. The exact mechanism is not clear; however, the use of unprotected boronic acid and the presence of a bidentate ligand appeared to be the key structural requirements for the transformation. The results suggest that base-promoted disproportionation of arylboronic acid with the assistance of the [*N*,*O*]-bidentate ligation of 1-(2-pyridinyl)-5-pyrazolone should take place and facilitate the formation of pyrazole diarylborinate. Experiments to obtain a deeper understanding of its mechanism are currently underway.

## 1. Introduction

Pyrazoles are important structural units that are frequently found in manypharmaceuticals, agrochemicals, and functional materials, as they serve as core scaffolds possessing a wide range of biological activities as well as synthetic templates for organic synthesis. Indeed, a large number of arylated pyrazoles have been synthesized and proven to be effective inhibitors of COX-2, p38 MAP kinase, and CDK2/Cyclin A [1]. In particular, we were involved in the tautomeric transformations of 5-pyrazolone derivatives for the synthesis of NADPH oxidase inhibitors [2,3,4,5].

In the course of the investigation, we unexpectedly found the formation of diarylborinate complexes, particularly for the case with 1-(2-pyridinyl)-5-pyrazolone derivatives and arylboronic acids. However, the four-coordinate boron compounds have been routinely prepared from the reaction of [*N*,*O*]-bidentate ligands with triarylboranes [6] and diarylborinic acids [7]. This is a subject of great interest since four-coordinate boron(III) complexes make them very useful as luminescent materials for organic electronics and photonics, and sensing and imaging probes for biomedical purposes [8]. To the best of our knowledge, the finding is a unique instance of the formation of diarylborinates via the direct employment of arylboronic acids.

Meanwhile, this observation raised important questions, such as: “Where does it come from”? Although many questions still remain to be answered, herein we focus on reporting our early understanding of the structural requirements of 1-(2-pyridinyl)-5-pyrazolone derivatives for the disproportionation of arylboronic acids.

## 2. Results and Discussion

**Scheme 1:**
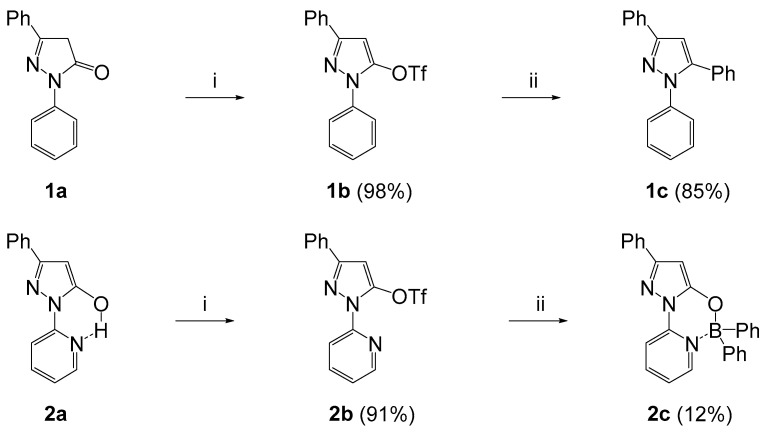
Reagents and conditions: (i), Tf_2_O, *i*-Pr_2_NEt, CHCl_3_, rt, 0.5 h; (ii), PhB(OH)_2_ (3 eq), PdCl_2_ (dppf) (8 mol%), dppf (4 mol%), K_3_PO_4_ (3 eq), dioxane, 100 °C, 1 h (for **1c**); 20 h (for **2c**).

The starting pyrazolones **1a** and **2a** were prepared by the condensation of ethyl benzoylacetate with the corresponding hydrazines, respectively [9]. Pyrazole triflates **1b** and **2b** were made by treating the corresponding pyrazolones with trifluoromethanesulfonic anhydride and *N*,*N*-diisopropylethylamine, where chloroform is the best choice to achieve excellent chemoselectivity and high yield [10]. Suzuki–Miyaura cross-coupling of **1b** furnished the corresponding 5-phenylpyrazole **1c** in 85% yield, as shown in Scheme 1. When **2b** was used, however, an unknown product was observed in small amounts along with prolonged reaction times. The spectroscopic data apparently show the introduction of two phenyl groups into the entity, but still it was difficult to elucidate the molecular structure.

**Scheme 2:**
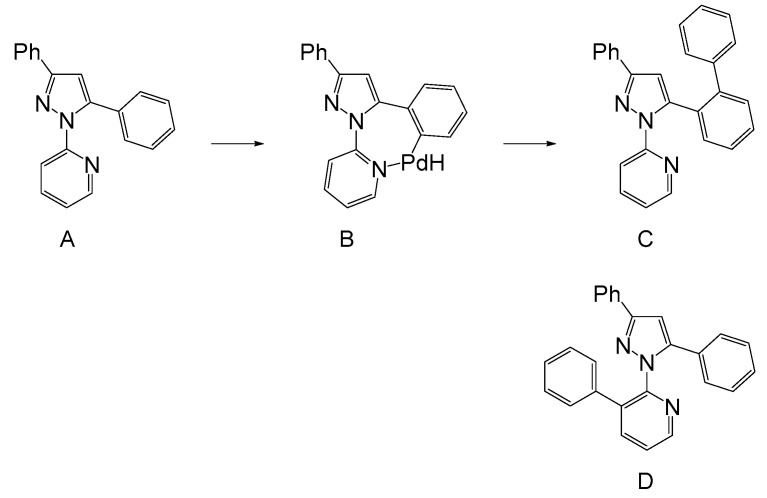
Initially proposed structures for **2c**.

Initially, we assumed **2c** would be a biphenylated product (C) resulting from the C-H activation of a Suzuki product (A from **2b**) as the pyridine ring is known to act as a directing group via the formation of a six membered palladacycle (B), as illustrated in Scheme 2 [11]. In addition, a diarylated pyrazole (D) is conceivable, in which the pyrazole nitrogen serves as a transformable directing group, as documented in the Pd-catalyzed sp^2^ C-H functionalization of *N*-arylpyrazole [12]. However, the C-H activation was not the case for **2c** since this does not explain the most abundant peak observed at *m*/*z =* 401.

Next, we examined whether palladium catalysis was likely to exert influence on the disproportionation/dimerization of arylboronic acid, taking into account the detection of a palladium(II) species with a diarylborinate anion in the coupling reaction of aryl bromides with arylboronic acids catalyzed by the palladium complex [13]. Thus, in order to figure out what was really responsible for this transformation, negative control experiments were performed by the removal of each component from the reaction vessel one by one. The results are summarized in Table 1.

When the reaction of **2b** was carried out without the palladium catalyst, this startlingly led to the same product (entry 1, Table 1). Furthermore, the use of the triflate was not a strict requirement for the transformation (entry 2). Subsequently, we ascertained that **2a** could be regenerated in the presence of a base from **2b**, presumably due to the hydrolytic instability of the triflate [14]. Another important feature was that there was no such indication from **1a** (entry 3). So far, nothing had changed when phenylboronic acid pinacol ester was used with **2a**, and the reaction was performed without the base (entries 4 and 5). To ensure a reliable formation of diarylborinate species, as the phenylboronic acid and the base were simply heated, we were able to isolate diphenylborinic acid **3a** and triphenylboroxin **3b** (entry 6). However, this could not be observed without the base. These observations demonstrate that base-induced disproportionation of boronic acids can also occur even without a chelate ligand, but will produce **3a** in lower grade. The final installation was undertaken to confirm the structure of **2c** (entry 7) by employing the reaction with diphenylborinic acid [15].

We explored the scope of the method by varying the boronic acids with different pyrazole substrates illustrated in Table 2. The reactions with 4-methoxy, 3-fluoro, 4-chloro, 4-bromo and 4-(*N*,*N*-diphenylamino)phenylboronic acid continually gave the products **2d**–**2h** in moderate yields, respectively. There were no noticeable effects with the electronic influence of boronic acid substituents. 2-Benzothienylboronic acid afforded the product **2i** at 41% yield without any issue, while 2-naphthylboronic acid afforded **2j** at 21% yield, suggesting that there was a steric effect in this transformation. Noticeably, boronic acids with sensitive functional groups such as ester (**2k**, 23%) and styrene (**2l**, 38%) were properly operated.

We then extended the scope of the pyrazole substrates with different substituents, such as 3-methyl and 4-trifluoromethylpyrazole, which gave **2m** (41%) and **2n** (23%), respectively. Meanwhile, there was no such indication again with *N*-(4-pyridinyl)pyrazole (**2o**), indicating that [*N*,*O*]-bidentation is inarguably the most fundamental feature. With these results, we can deduce that *N*-(2-pyridinyl)pyrazole is stabilized by an intramolecular hydrogen bond and, thus, exists in a *syn*-periplanar orientation, which is well adjusted to accommodate an incoming boronic acid.

We primarily revealed that palladium catalysis has no role in the formation of the product. The use of unprotected boronic acid and a base is vital, and the presence of [*N*,*O*]-bidentate ligand seems to be the crucial structural requisite for this transformation. Previously, we reported that **1a** remains as it stands, whereas **2a** exclusively exists in the enol-form [2], and the X-ray crystal structure clearly shows that the carbonyl oxygen and pyridine nitrogen adopt an almost *syn*-periplanar arrangement that is capable of accommodating intramolecular hydrogen bonding.

**Figure 1:**
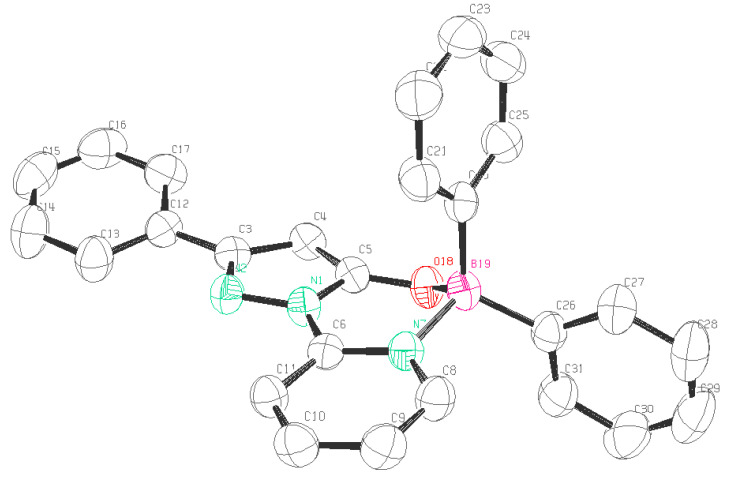
The crystal structure of **2c**.

Pleasingly, we were able to obtain a single crystal and determine the structure of **2c** as a six-membered pyrazole diphenylborinate complex (Figure 1). Single crystals suitable for X-ray diffraction were prepared by slow evaporation of a solution in ethyl acetate at room temperature. It is noteworthy that the crystal structure exhibited a pseudo-tetrahedral geometry around the boron center linked to two phenyl groups and with a [*N*,*O*]-bidentate chelating ligand. In addition, there was a broad singlet centered at 7.81 ppm in the ^11^B NMR spectrum of **2c**.

Single-crystal X-ray diffractions were measured on a Bruker APEX-II CCD diffractometer equipped with a monochromatic Mo-Kα radiation (λ = 0.71073 Å). The data were collected at a low temperature of 100 K by the φ-ω scan method. The collected data were integrated using Bruker-SAINT software and an absorption correction was not applied. The structure was solved and refined through the least-squares method with the SHELXT and SHELXL program, respectively. All the non-hydrogen atoms were refined anisotropically and hydrogen atoms were placed in calculated positions. Table 3 presents the crystallographic data and structural refinements. Atomic coordinates and crystallographic parameters for **2c** were deposited at the Cambridge Crystallographic Data Centre (DOI: 10.5517/ccdc.csd.cc28yrgg, CCDC number: 2113966).

**Scheme 3:**
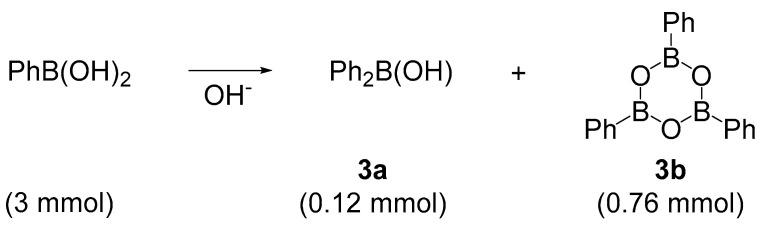
Disproportionation of phenylboronic acid (entry 6, Table 1).

Although a number of possible explanations can be advanced for such a unique transformation, we probed that the disproportionation of arylboronic acid could be induced by the base, with or without a bidentate ligand. Firstly, when 3 mmol of phenylboronic acid and 3 mmol of potassium phosphate were simply heated without ligand, we were able to isolate 0.12 mmol of diphenylborinic acid **3a** along with triphenylboroxin **3b**, as depicted in Scheme 3. This observation demonstrates that base-induced disproportionation of boronic acids is possible even without a chelate ligand, but will produce **3a** with a lower efficiency. Meanwhile, in the presence of a bidentate ligand, the base-promoted disproportionation of arylboronic acid was accelerated, and thus, the formation of pyrazole diarylborinate occurred (entries 2 and 6, Table 1).

**Scheme 4:**
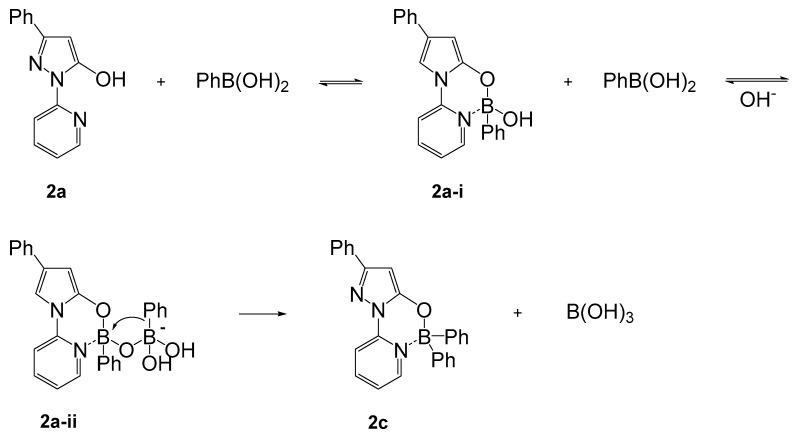
A plausible mechanism for the formation of **2c** induced by [*N*,*O*]-bidentate ligation.

Based on the experimental considerations, we propose a plausible mechanism for the formation of the four-coordinate boron species facilitated by the assistance of the [*N*,*O*]*-*bidentate ligand, which enabled the aryl group migration between boronic acids, as illustrated in Scheme 4. We were aware that **2a** as the enol could be modulated by its complexation to phenylboronic acid (**2a**–**i**). Accordingly, a recent report revealed that boronic acids can disrupt the intramolecular proton transfer fluorescence through complexation with 10-hydroxybenzo[*h*]quinolone by disrupting the intramolecular hydrogen bond [16]. Considering the thermodynamics of boronic acids, entropically favorable dimeric anhydride or trimeric aggregate might be involved in this transformation [17]. Particularly, the Petasis borono–Mannich reaction [18] has been extensively studied, in which the boronic acids act as organic group donors under metal-free transition conditions, and protodeboronation [19] and boron-to-heteroatom migration [20] have also been utilized via boronate complexes derived from different types of boronic anhydride species. The base may be required to drive the initial equilibrium sufficiently toward the ‘ate’ complex (**2a-ii**) so that boron-to-boron migration is feasible. The ‘ate’ complex is believed to be able to transfer the organic group from the anionic boron center onto a nearby electron-deficient sp^2^ boron through a boronic anhydride assembly.

## 3. Materials and Methods

### 3.1. Generals

All solvents and reagents were purchased from commercial sources and used as received without further purification, unless otherwise stated. Tripotassium phosphate was crushed in mortar and dried at 70 °C in oven overnight and used. Reactions were monitored by thin layer chromatography carried out on S-2 0.25 mm E. Merck silica gel plates (60F-254, Darmstadt, Germany) using UV light as the visualizing agent and an acidic mixture of anisaldehyde or a ninhydrin solution in ethanol and heat as developing agents. E. Merck silica gel (60, particle size 0.040–0.063 mm) was used for flash column chromatography. All yields were calculated from isolated products. All NMR spectra were recorded on Bruker AV-500 instrument. ^1^H and ^13^C NMR spectra were referenced internally to the residual undeuterated chloroform (δ_H_ = 7.26 ppm and δ_C_ = 77.0 ppm). ^11^B NMR spectra were referenced externally to BF_3_.OEt_2_. The ^11^B NMR experiments were done with quartz NMR tubes (Wilmad). The NMR data were analyzed using MNova 10.0 processing software (version: MNova 14.1.0) (Mestrelab Research). The following abbreviations are used to designate multiplicities: s = singlet, d = doublet, t = triplet, q = quartet, quint = quintet, m = multiplet, br s = broad singlet. Chemical shifts are reported in ppm and coupling constants are in Hertz (Hz). High resolution mass spectra using Electronic Ionization (HRMS-EI) were recorded on Joel JMS-700 mass spectrometer. The data for X-ray structure determination were collected on Bruker SMART Apex II X-ray diffractometer equipped with graphite-monochromated MoKα radiation (λ = 0.71073 Å). The ^1^H and ^13^C NMR spectra for all compounds **2c**–**2n** prepared in this study are in Supplementary Materials.

### 3.2. Representative Procedure for the Synthesis of Four-Coordinate Boron Complexes

A solution of **2a** (237.3 mg, 1.0 mmol), PhB(OH)_2_ (365.8 mg, 3.0 mmol) and K_3_PO_4_ (636.8 mg, 3.0 mmol) in 1,4-dioxane (10 mL) was heated to reflux at 100 °C for 20 h. Subsequently, the solvent was removed by evaporation and the crude was extracted with EtOAc (2 × 10 mL). The organic layer was washed with saturated NaHCO_3_ and brine, and dried over anhydrous sodium sulfate. The solvent was removed under vacuum and the residue was purified by column chromatography on silica gel (hexane/EtOAc = 7/1) to give **2c** (148.5 mg, 37% yield).

2-(5-((Diphenylboryl)oxy)-3-phenyl-1*H*-pyrazol-1-yl)pyridine (**2c**). 

Yield: 37%; ^1^H NMR (500 MHz, CDCl_3_): δ_H_ 8.18–8.04 (m, 2H), 7.93 (dd, *J* = 6.2, 1.6 Hz, 1H), 7.87–7.78 (m, 2H), 7.44–7.34 (m, 3H), 7.32–7.21 (m, 11H), 5.98 (s, 1H) ppm; ^13^C NMR (125 MHz, CDCl_3_): δ_C_ 157.06, 156.6, 147.0, 143.0, 142.1, 133.0, 132.5, 129.1, 128.6, 127.6, 127.2, 126.0, 119.3, 113.4, 86.6 ppm; ^11^B NMR (160 MHz, CDCl_3_): δ_B_ 7.81 ppm; HRMS-EI *m*/*z* [M]^+^ calcd for C_26_H_20_N_3_OB, 401.1699, found 401.1692.

Atomic coordinates and crystallographic parameters for **2c** has been deposited at the Cambridge Crystallographic Data Center (DOI: 10.5517/ccdc.csd.cc28yrgg, CCDC number: 2113966). These data can be obtained free of charge from the Cambridge Crystallographic Data Center via www.ccdc.cam.ac.uk/data_request/cif (accessed on 9 November 2021).

2-(5-((Bis(4-methoxyphenyl)boryl)oxy)-3-phenyl-1*H*-pyrazol-1-yl)pyridine (**2d**).

Yield: 45%; ^1^H NMR (500 MHz, CDCl_3_): δ_H_ 8.15 (m, 2H), 7.96 (d, *J* = 5.4 Hz, 1H), 7.85 (d, *J* = 7.05 Hz, 2H), 7.45–7.38 (m, 3H), 7.26–7.22 (m, 5H), 6.84 (d, *J* = 8.45 Hz, 4H), 5.98 (s, 1H), 3.79 (s, 6H) ppm; **^13^C** NMR (125 MHz, CDCl_3_): δ_C_ 158.9, 156.9, 156.7, 147.0, 142.8, 142.0, 134.2, 132.6, 129.1, 128.6, 126.0, 119.2, 113.3, 113.2, 86.5, 55.0 ppm; HRMS (EI) *m*/*z* [M + H]^+^ calcd for C_28_H_24_N_3_O_3_B, 461. 1911, found 461. 1911.

2-(5-((Bis(3-fluorophenyl)boryl)oxy)-3-phenyl-1*H*-pyrazol-1-yl)pyridine (**2e**). 

Yield: 27%; ^1^H NMR (500 MHz, CDCl_3_): δ_H_ 8.16 (dd, *J* = 15.8, 7.7 Hz, 2H), 7.89 (dd, *J* = 16.3, 6.1 Hz, 3H), 7.44 (dt, *J* = 13.8, 6.9 Hz, 3H), 7.28 (dd, *J* = 13.6, 7.1 Hz, 3H), 7.06 (d, *J* = 7.2 Hz, 2H), 6.97 (dd, *J* = 14.9, 9.1 Hz, 4H), 6.04 (s, 1H) ppm; ^13^C NMR (126 MHz, CDCl_3_): δ_C_ 163.9, 161.98, 157.3, 156.2, 146.8, 143.5, 141.7, 132.3, 129.37 (d, *J* = 7.6 Hz), 128.68, 128.37 (d, *J* = 1.9 Hz), 126.1, 119.59, 119.2 (d, *J* = 18.2 Hz), 114.3 (d, *J* = 21.0 Hz), 114.18, 114.0, 113.7, 86.80 ppm; HRMS-EI *m*/*z* [M]^+^ calcd for, C_26_H_18_BF_2_N_3_O 437.1511, found 437.1509.

2-(5-((Bis(4-chlorophenyl)boryl)oxy)-3-phenyl-1*H*-pyrazol-1-yl)pyridine (**2f**). 

Yield: 31%; ^1^H NMR (500 MHz, CDCl_3_): δ_H_ 8.23–8.08 (m, 2H), 7.86 (d, *J* = 6.6 Hz, 3H), 7.52–7.38 (m, 3H), 7.25 (dd, *J* = 27.6, 8.1 Hz, 9H), 6.01 (s, 1H) ppm; ^13^C NMR (126 MHz, CDCl_3_): δ_C_ 157.3, 156.3, 146.9, 143.4, 141.6, 134.3, 133.5, 132.3, 129.3, 128.7, 127.9, 126.1, 119.6, 113.7, 86.7 ppm; HRMS-EI *m*/*z* [M]^+^ calcd for,C_26_H_18_BCl_2_N_3_O 469.0920, found 469.0927.

2-(5-((Bis(4-bromophenyl)boryl)oxy)-3-phenyl-1*H*-pyrazol-1-yl)pyridine (**2g**). 

Yield: 35%; ^1^H NMR (500 MHz, CDCl_3_): δ_H_ 8.26–8.11 (m, 2H), 7.93–7.79 (m, 3H), 7.49–7.37 (m, 7H), 7.34–7.27 (m, 4H), 7.15 (d, *J* = 8.2 Hz, 4H), 5.99 (s, 1H) ppm; ^13^C NMR (125 MHz, CDCl_3_): δ_C_ 157.3,156.2, 143.4, 141.6, 134.6, 130.8, 129.3, 128.7, 126.1, 119.5, 113.7, 86.7, 77.3, 77.0, 76.8 ppm; HRMS-EI *m*/*z* [M]^+^ calcd for,C_26_H_18_BBr_2_N_3_O 566.9910, found 566.9918.

4,4’-(((3-Phenyl-1-(pyridin-2-yl)-1*H*-pyrazol-5-yl)oxy)boranediyl)bis(*N,N*-diphenylaniline) (**2h**). 

Yield: 27%; ^1^H NMR (500 MHz, CDCl_3_): δ_H_ 8.11 (dd, *J* = 14.0, 7.7 Hz, 1H), 8.06–7.96 (m, 1H), 7.86 (d, *J* = 7.8 Hz, 1H), 7.54–7.32 (m, 2H), 7.21 (ddd, *J* = 19.5, 10.9, 6.7 Hz, 8H), 7.07 (d, *J* = 8.3 Hz, 4H), 7.02–6.84 (m, 5H), 5.98 (d, *J* = 1.3 Hz, 1H) ppm; ^13^C NMR (126 MHz, CDCl_3_): δ_C_ 156.7, 147.9, 146.7, 142.9, 142.1, 133.8, 129.2, 129.0, 128.6, 126.0, 124.1, 123.2, 122.3, 119.2, 113.3, 86.6 ppm; HRMS-EI *m*/*z* [M]^+^ calcd for C_50_H_38_N_5_OB, 735.3169, found 735.3177.

2-(5-((Bis(benzo[*b*]thiophen-2-yl)boryl)oxy)-3-phenyl-1*H*-pyrazol-1-yl) pyridine (**2i**). 

Yield: 41%; ^1^H NMR (300 MHz, CDCl_3_): δ_H_ 8.52 (dd, *J* = 1.26, 8.67 Hz, 1H), 8.29–8.19 (m, 2H), 7.94–7.86 (m, 4H), 7.80–7.76 (m, 2H), 7.66 (dd, *J* = 0.51, 7.08 Hz, 1H), 7.49–7.37 (m, 3H), 7.35–7.24 (m, 4H), 7.71 (s, 2H), 6.42 (s, 1H) ppm; ^13^C NMR (125 MHz, CDCl_3_): δ_C_ 156.3, 155.1, 146.6, 145.7, 142.3, 142.2, 141.2, 132.2, 129.9, 129.2, 129.1, 126.3, 124.4, 124.3, 123.8, 122.7, 122.5, 113.8, 87.3 ppm; HRMS (EI) *m*/*z* [M + H]^+^ calcd for C_30_H_20_N_3_OS_2_B, 513. 1141, found 513.1141.

2-(5-((Di(naphthalen-2-yl)boraneyl)oxy)-3-phenyl-1*H*-pyrazol-1-yl)pyridine (**2j**). 

Yield: 21%; ^1^H NMR (500 MHz, CDCl_3_): δ_H_ 8.18 (d, *J* = 8.4 Hz, 1H), 8.10 (t, *J* = 7.3 Hz, 1H), 8.02 (d, *J* = 5.1 Hz, 1H), 7.84 (dd, *J* = 12.7, 7.1 Hz, 6H), 7.77–7.67 (m, 4H), 7.61 (d, *J* = 8.1 Hz, 2H), 7.49–7.36 (m, 7H), 7.22 (t, *J* = 6.1 Hz, 1H), 6.07 (s, 1H) ppm; ^13^C NMR (126 MHz, CDCl_3_): δ_C_ 157.2, 156.7, 147.1, 143.2, 142.1, 133.2, 132.8, 132.5, 130.6, 129.2, 128.6, 128.1, 127.6, 127.0, 126.1, 125.5, 119.4, 113.5, 86.7 ppm; HRMS-EI *m*/*z* [M]^+^ calcd for,C_34_H_24_BN_3_O 501.2012, found 501.2019.

Dimethyl 4,4’-(((3-phenyl-1-(pyridin-2-yl)-1*H*-pyrazol-5-yl)oxy)boranediyl)dibenzoate (**2k**). 

Yield: 23%; ^1^H NMR (500 MHz, CDCl_3_): δ_H_ 8.17–8.04 (m, 2H), 7.93 (dd, *J* = 6.2, 1.6 Hz, 1H), 7.87–7.78 (m, 2H), 7.44–7.34 (m, 2H), 7.32–7.21 (m, 11H), 5.98 (s, 1H), 3.87 (s, 6H) ppm; ppm; ^13^C NMR (125 MHz, CDCl_3_): δ_C_ 165.9, 160.0, 150.4, 145.1, 142.8, 139.3, 133.3, 130.1, 129.9, 129.2, 127.5, 121.4, 112.4, 86.8, 51.5 ppm; HRMS-EI *m*/*z* [M]^+^ calcd for C_30_H_24_N_3_O_5_B, 517.1809, found 517.1813.

2-(5-((Di((*E*)-styryl)boraneyl)oxy)-3-phenyl-1*H*-pyrazol-1-yl)pyridine (**2l**). 

Yield: 38%; ^1^H NMR (300 MHz, CDCl_3_): δ_H_ 8.31 (d, *J* = 5.73 Hz, 1H), 8.16 -8.06 (m, 2H), 7.88–7.85 (m, 2H), 7.46–7.35 (m, 6H), 7.32–7.27 (m, 6H), 7.20–7.15 (m, 2H), 6.71–6.63 (m, 4H), 5.96 (s, 1H) ppm; ^13^C NMR (125 MHz, CDCl_3_): δ_C_ 156.9, 156.1, 146.8, 142.8, 141.3, 139.2, 139.0, 132.6, 129.1, 128.6, 128.3, 127.1, 126.3, 126.1, 119.7, 113.3, 86.35ppm; HRMS (EI) *m*/*z* [M + H]^+^ calcd for C_30_H_24_N_3_OB, 453.2012, found 453.2017.

2-(5-((Diphenylboraneyl)oxy)-3-methyl-1*H*-pyrazol-1-yl)pyridine (**2m**). 

Yield: 41%; ^1^H NMR (500 MHz, CDCl_3_): δ_H_ 8.07–8.04 (m, 2H), 7.91 (dd, *J* = 6.2, 1.6 Hz, 1H), 7.32–7.21 (m, 11H), 5.98 (s, 1H), 2.34 (s, 3H) ppm; ^13^C NMR (125 MHz, CDCl_3_): δ_C_ 159.9, 150.4, 149.7, 139.3, 138.5, 133.4, 128.7, 121.4, 112.4, 91.2, 16.5 ppm; HRMS-EI *m*/*z* [M]^+^ calcd for C_21_H_18_N_3_OB, 339.1543, found 339.1552.

2-(5-((Diphenylboraneyl)oxy)-3-methyl-4-(trifluoromethyl)-1*H*-pyrazol-1-yl)pyridine (**2n**). 

Yield: 23%; ^1^H NMR (500 MHz, CDCl_3_): ^1^H NMR (500 MHz, CDCl_3_): δ_H_ 8.21–8.13 (m, 1H), 8.10 (d, *J* = 8.3 Hz, 1H), 8.02–7.95 (m, 1H), 7.41–7.34 (m, 1H), 7.32–7.21 (m, 10H), 2.12 (s, 3H).^13^C NMR (126 MHz, CDCl_3_): δ_C_ 153.8, 146.5, 143.5, 142.5, 132.8, 127.7, 127.4, 122.2, 120.8, 120.0, 113.6, 96.3, 5.78 ppm; HRMS-EI *m*/*z* [M]^+^ calcd for, C_22_H_17_BF_3_N_3_O 407.1417, found 407.1411.

## 4. Conclusions

In this study, we found a simple, mild, transition metal-free method for the preparation of four-coordinate organoboron complexes, and also discussed on the key components and the structural requirements that enable such a boron-to-boron migration. While the use of unprotected boronic acid and a base is essential, the presence of [*N*,*O*]-bidentate ligand appeared to be the key structural requirements for this transformation. Based on the control experiments, the results support that four-coordinate boron species derived from the [*N*,*O*]-bidentate ligand **2a** favor the formation of diarylborinic acid and/or disproportionation of arylboronic acid via the action of the base. The *syn*-periplanar arrangement of the [*N*,*O*]-bidentate ligand was found to be crucial. It was well organized to accommodate an incoming boronic acid, and thus, to enable to aryl group migration between boronic acids, presumably via a boronic anhydride species. Overall, the present method is particularly important in preparing four-coordinate organoboron species to ensure a completely efficient assembly of multi-component structures in a single operation. Experiments to obtain a deeper understanding of its mechanism and applications are currently underway.

## Figures and Tables

**Table 1:** Control experiments.

Entry	Substrate ^a^	Reaction Conditions ^b^	Product (Yield, %)
1	**2b**	PhB(OH)_2_, K_3_PO_4_, dioxane	**2c** (29)
2	**2a**	PhB(OH)_2_, K_3_PO_4_, dioxane	**2c** (37)
3	**1a**	PhB(OH)_2_, K_3_PO_4_, dioxane	- ^c^
4	**2a**	PhB(OCMe_2_CMe_2_O), K_3_PO_4_, dioxane	- ^c^
5	**2a**	PhB(OH)_2_, dioxane	- ^c^
6	- ^d^	PhB(OH)_2_, K_3_PO_4_, dioxane	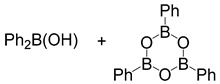 **3a** (4%) **3b** (26%) ^e^
7	**2a**	**3a**, EtOH	**2c** (81%)

^a^ The structures shown in Scheme 1. ^b^ Unless otherwise indicated, the reaction conditions are as follows: substrate (1.0 mmol), boron reagent (3 eq), base (3 eq), solvent (10 mL), 100 °C, 20 h. ^c^ No reaction observed. ^d^ Without substrate. ^e^ See, Scheme 3.

**Table 2:** Scope of boronic acids and pyrazole substrates ^a^.

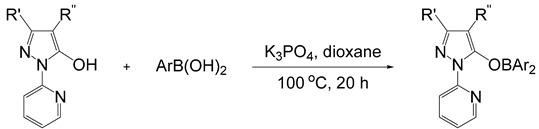
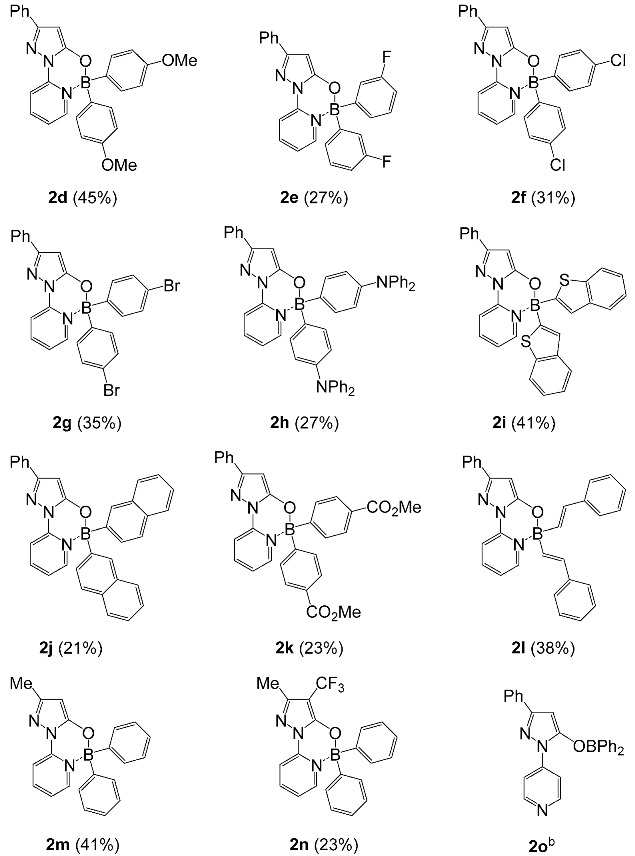

^a^ Reaction conditions: pyrazole substrate (1 mmol), ArB(OH)_2_ (3 eq), K_3_PO_4_ (3 eq), dioxane (10 mL), 100 °C, 20 h; where R’ = Ph, Me, R” = H, CF_3_. ^b^ Not observed.

**Table 3:** Crystallographic data of **2c**.

Crystal Data		
C_26_H_20_BN_3_O	* c* = 11.2162 (1) Å	* Z* = 2
*Mr* = 401.26	α = 78.966 (1)^o^	Mo Kα radiation
Triclinic, P1	β = 81.795 (1)^o^	μ = 0.08 mm^−1^
*a* = 9.7309 (1) Å	γ = 79.993 (1)^o^	*T* = 296 K
*b* = 9.8830 (1) Å	*V* = 1035.91 (2) Å^3^	0.44 × 0.31 × 0.23 mm
**Data Collection**		
Bruker APEXII CCD diffractometer		5044 independent reflections
19,866 measured reflections		3923 reflections with *I* > 2σ(*I*)
**Refinement**		
*R*[*F*^2^ > 2σ(*F*^2^)] = 0.041		280 parameters
*wR*(*F*^2^) = 0.106		H-atom parameters constrained
*S* = 1.03		Δρ_max_ = 0.21 *e* Å^−3^
5044 reflections		Δρ_min_ = −0.19 *e* Å^−3^

## Data Availability

The data presented in this study are available on request from the corresponding authors.

## Supplementary Materials

The following are available online. Online supplementary information contains ^1^H and ^13^C NMR spectra for all compounds **2c**–**2n** prepared in this study (**Figures S1**, **S2** and **S4**–**S25**), and 11B NMR of **2c** (**Figure S3**). CCDC 2113966 contains atomic coordinates and crystallographic parameters for **2c** and these data can be obtained free of charge from the Cambridge Crystallographic Data Centre via http://www.ccdc.cam.ac.uk/conts/retrieving.html, accessed on 2 November 2021.

## Sample Availability

Samples of the compounds are available from the authors.
