# Supplementary material for: Structural Requirements of 1-(2-Pyridinyl)-5-pyrazolones for Disproportionation of Boronic Acids
Source: Molecules. 2021 Nov 11;26(22):6814. doi: 10.3390/molecules26226814 (PMC8623043; doi:10.3390/molecules26226814)
Supplement: Supplementary file 1 [file molecules-26-06814-s001.zip › molecules-1449344-supplementary.pdf]

Supplementary Materials

Structural Requirements of 1-(2-Pyridinyl)-5-pyrazolones for Disproportionation of Boronic Acids

Joungmo Cho [1,†], Venkata Subbaiah Sadu [2,†], Yohan Han [1], Yunsoo Bae [3], Hwajeong Lee [4] and Kee-In Lee [1,2,*]

[1] Korea Research Institute of Chemical Technology, Daejeon 34114, Korea; jmcho@krict.re.kr (J.C.); yhhan@krict.re.kr (Y.H.)
[2] Molecules & Materials Co., Ltd. B-219 Daeduck BIZ Center, Daejeon 34013, Korea; subboo32@outlook.com
[3] Department of Life Science, Ewha Womans University, Seoul 03760, Korea; baeys@ewha.ac.kr
[4] Graduate School of Pharmaceutical Sciences, Ewha Womans University, Seoul 03760, Korea; hwalee@ewha.ac.kr
* Correspondence: kilee@krict.re.kr
† These authors contributed equally to this work.

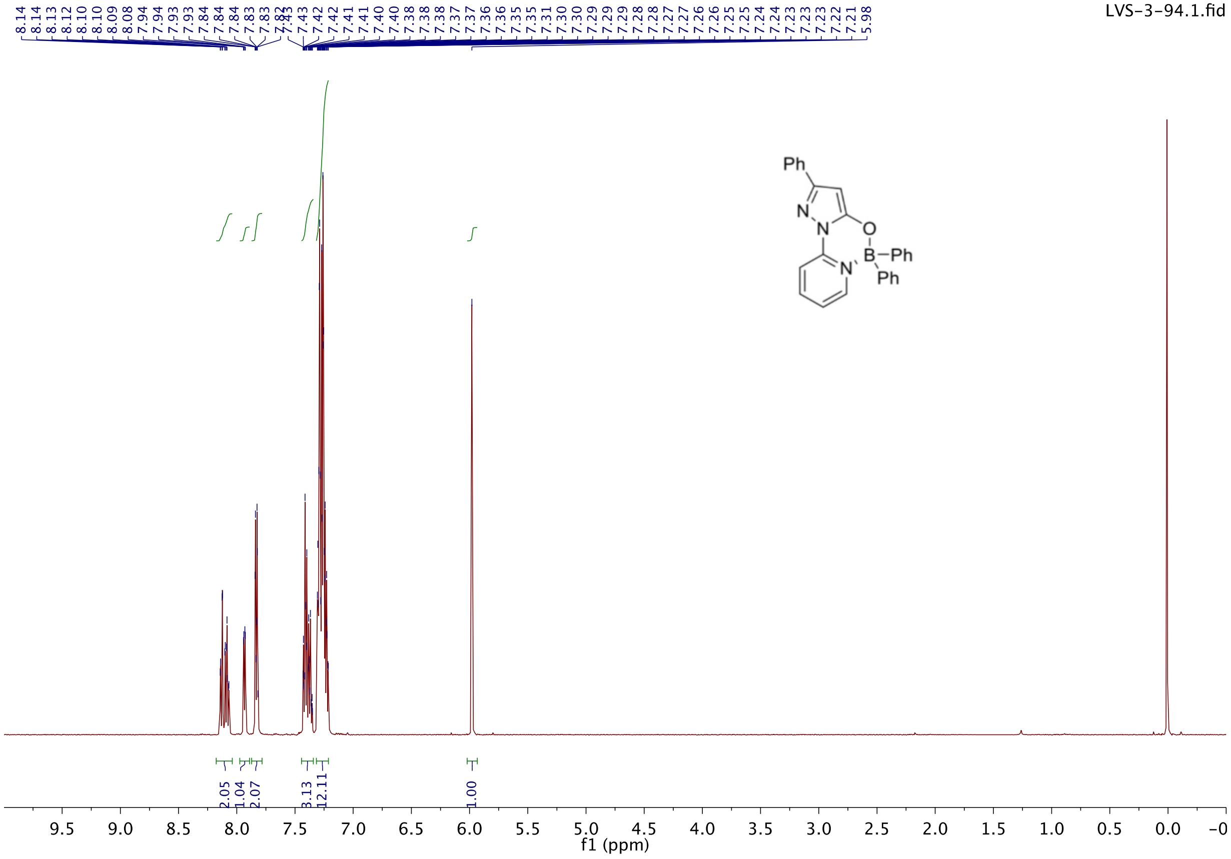

Figure S1. ^{1}H NMR of **2c** (500 MHz, $CDCl_3$)

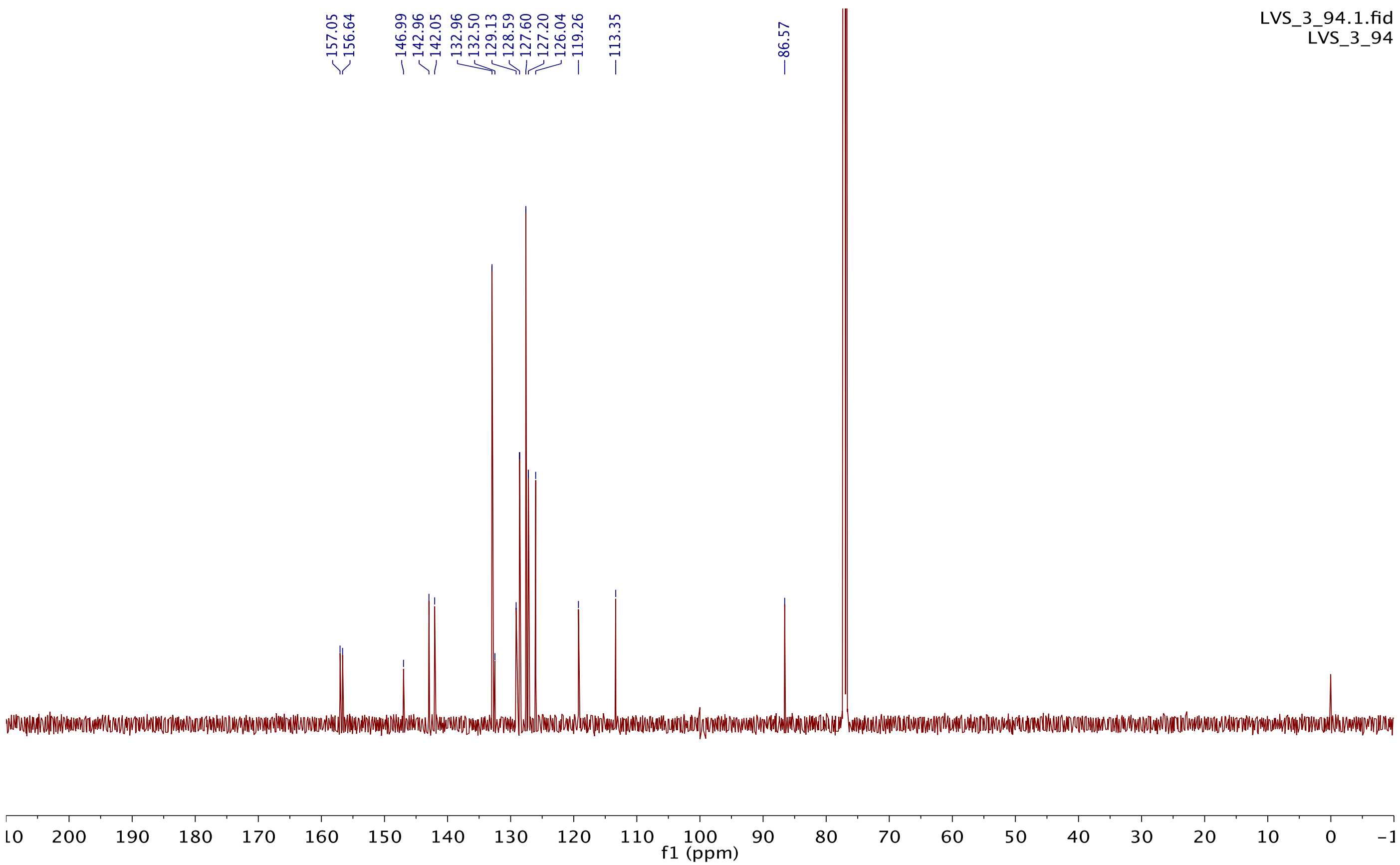

Figure S2. ^{13}C NMR of **2c** (125 MHz, $CDCl_3$)

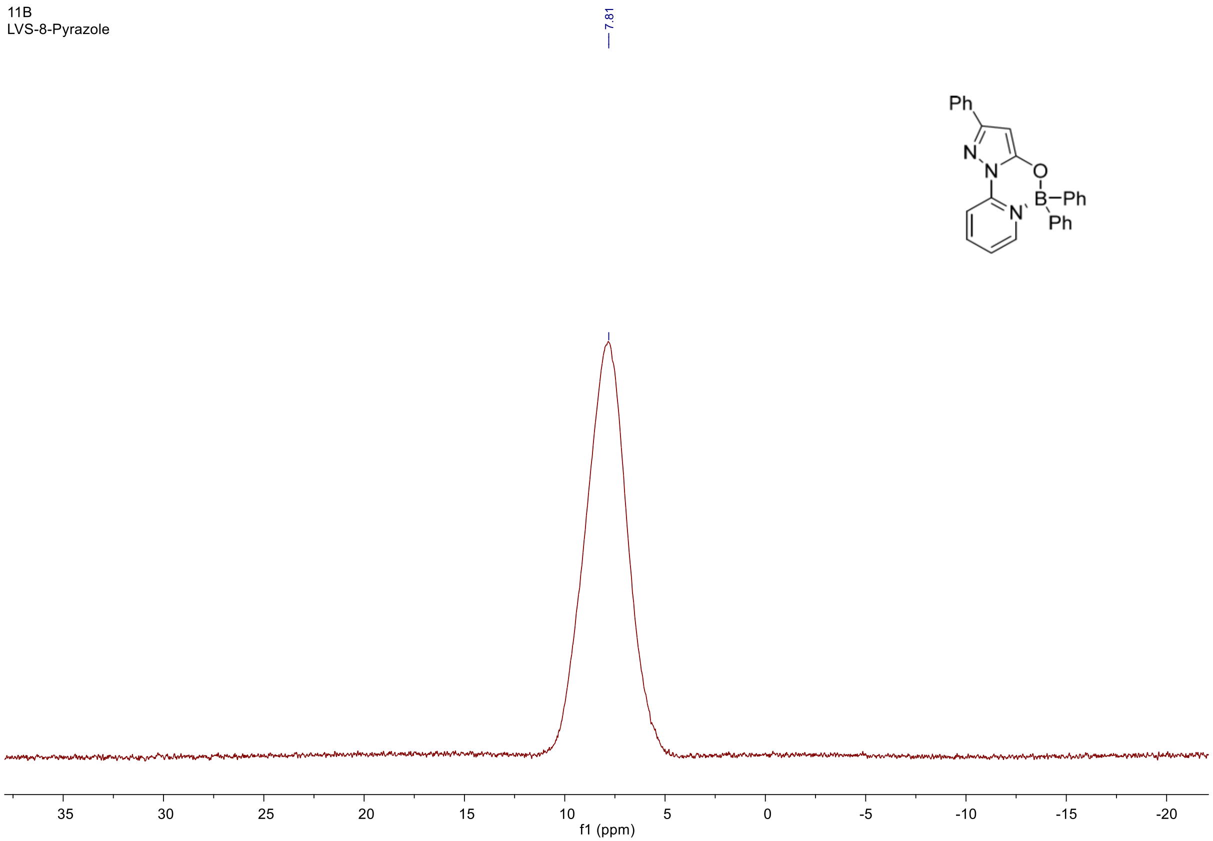

Figure S3. ^{11}B NMR of **2c** (160 MHz, $CDCl_3$)

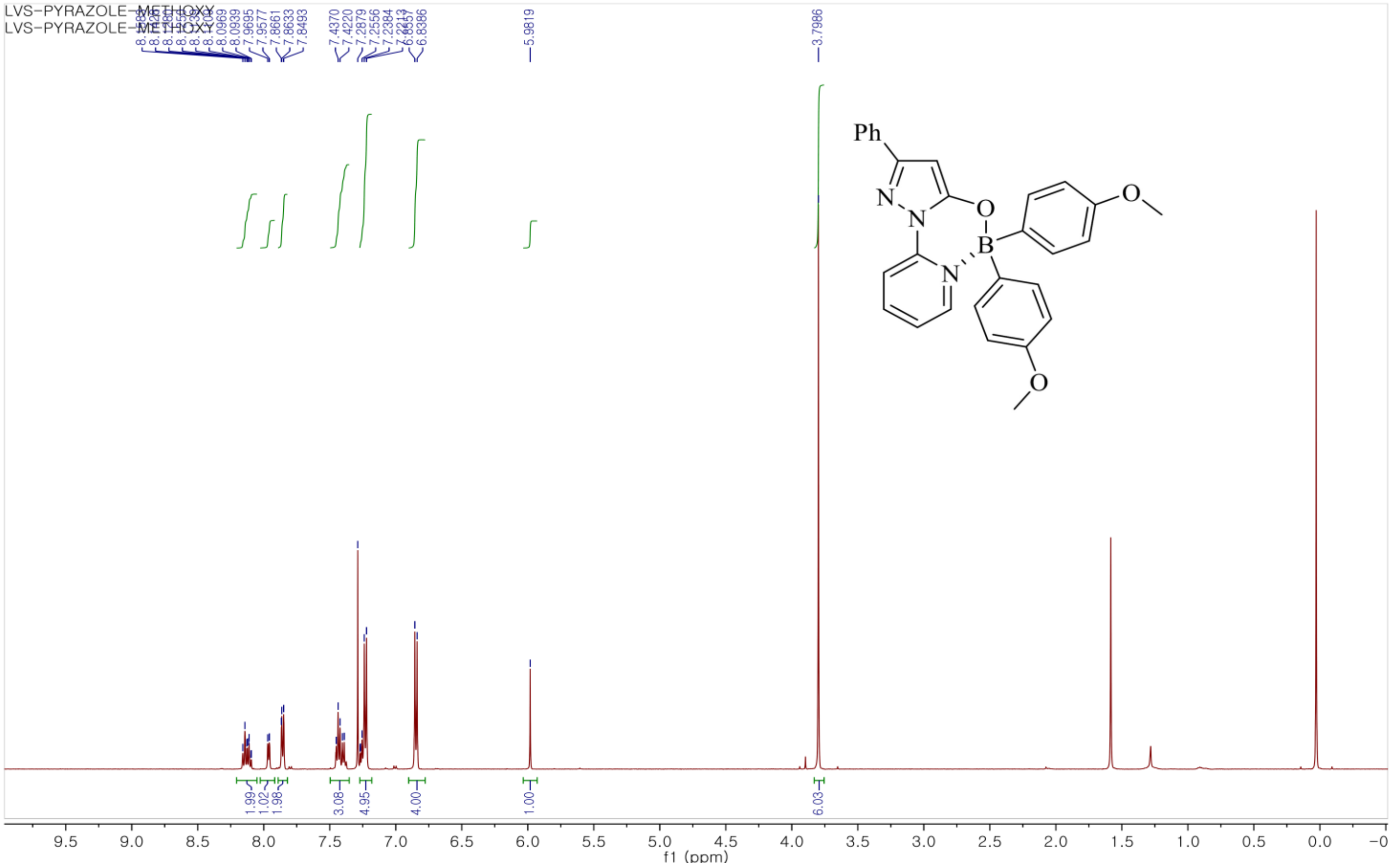

Figure S4. ^{1}H NMR of **2d** (500 MHz, $CDCl_3$)

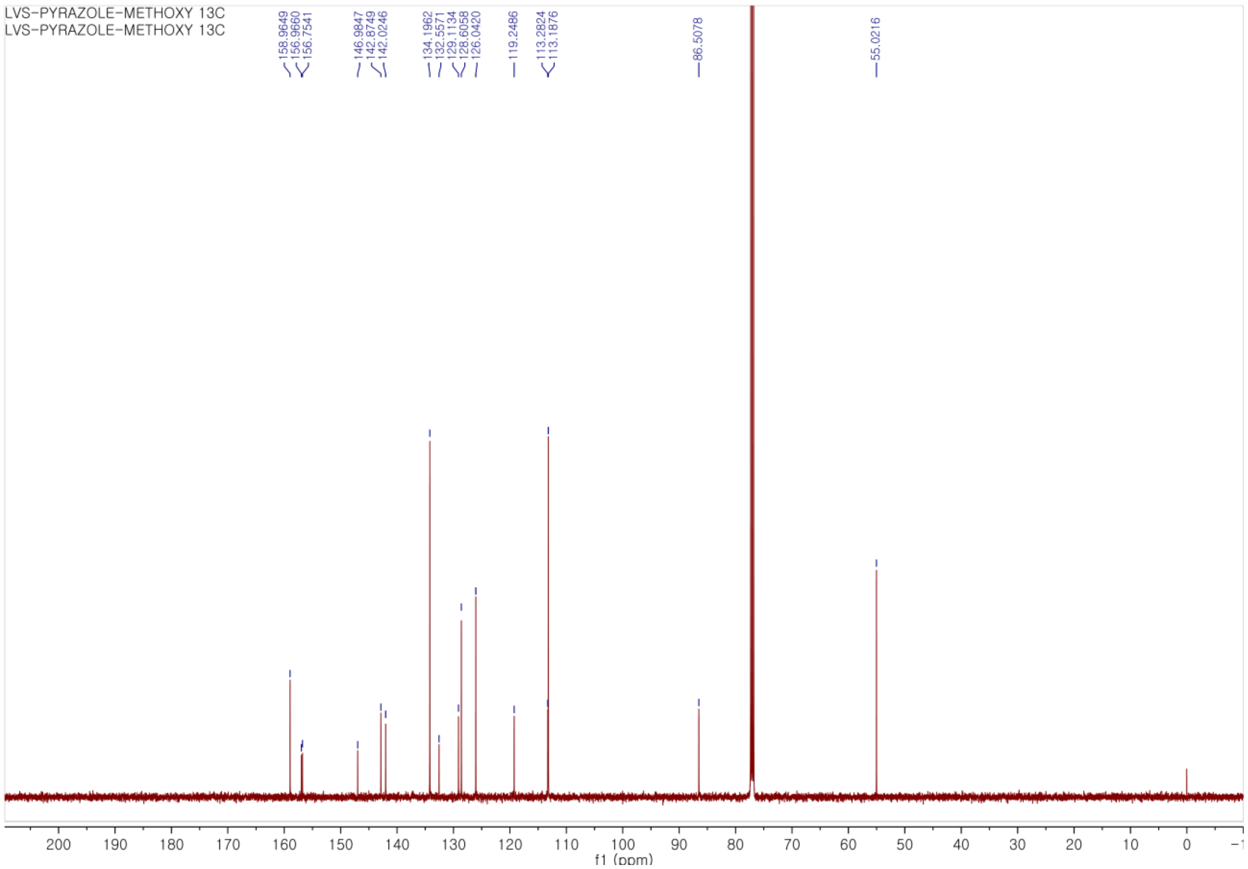

Figure S5. ^{13}C NMR of **2d** (125 MHz, $CDCl_3$)

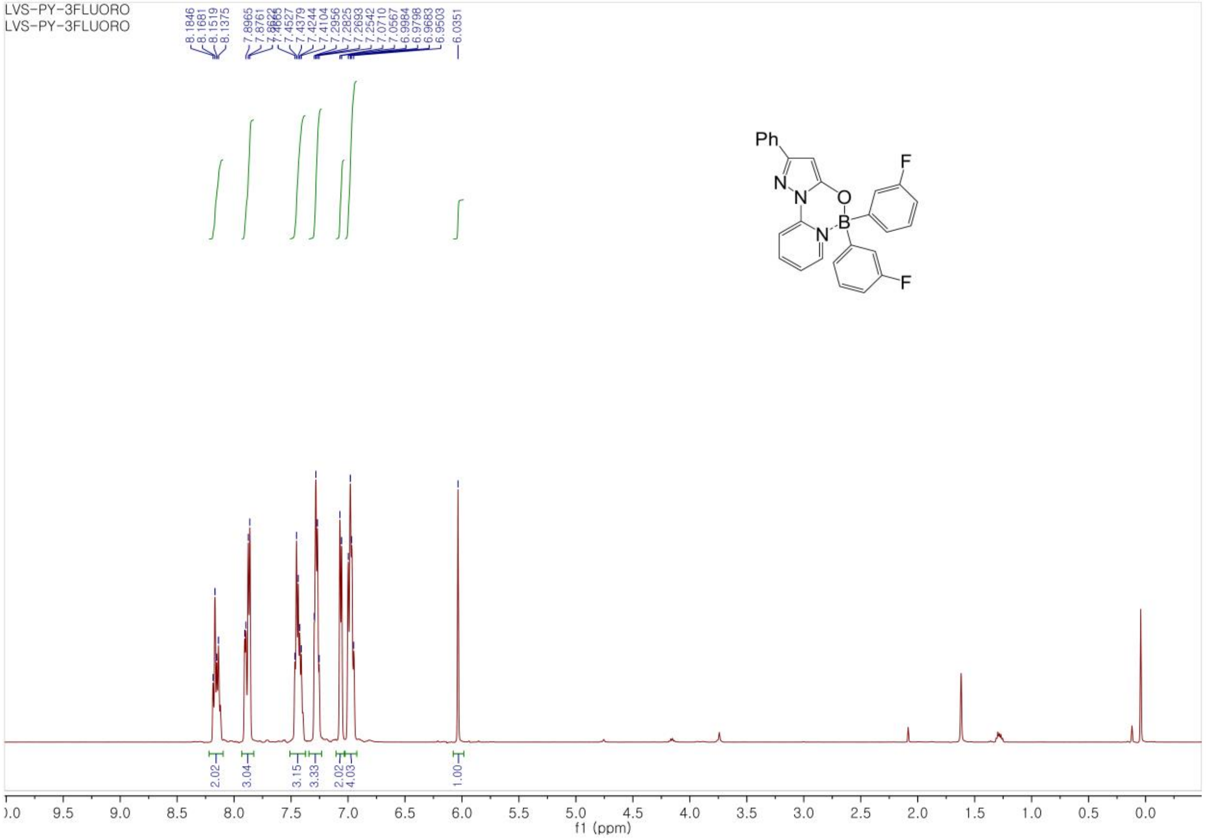

Figure S6. ^{1}H NMR of **2e** (500 MHz, $CDCl_3$)

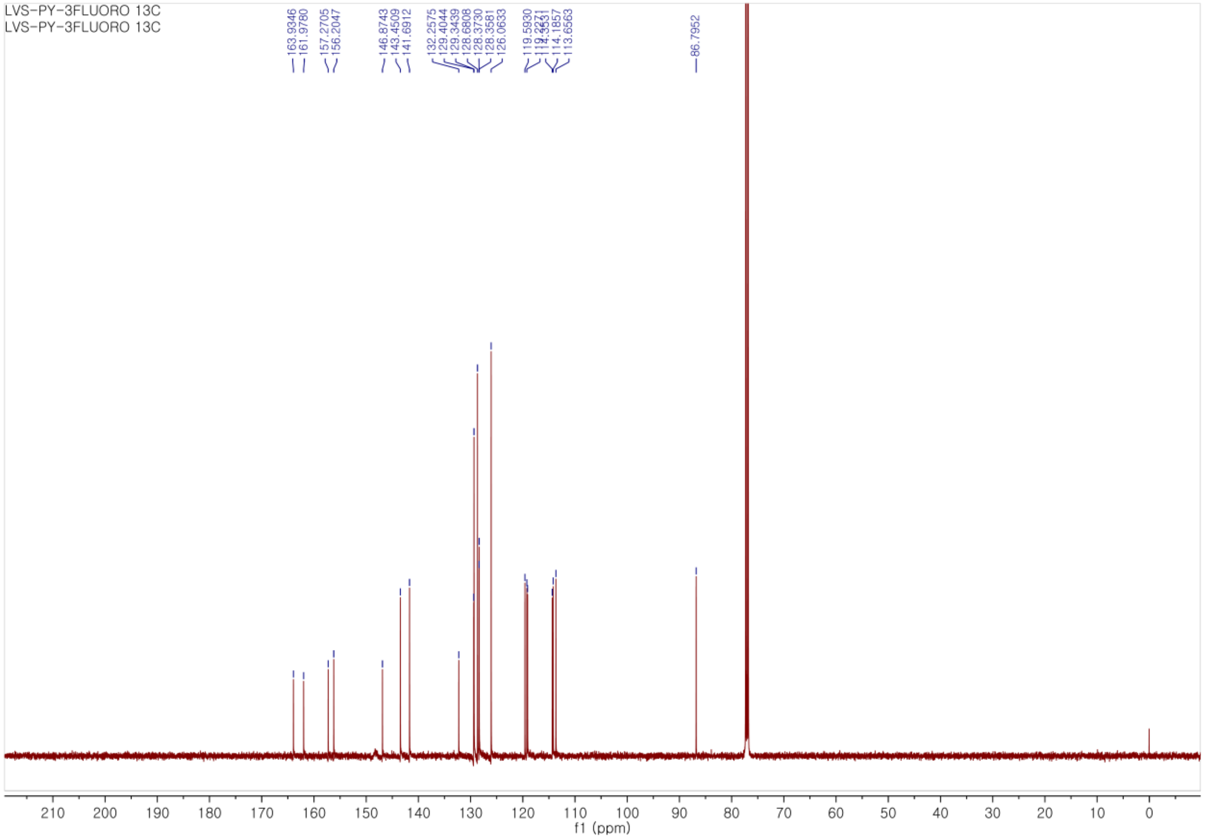

Figure S7. ^{13}C NMR of **2e** (125 MHz, $CDCl_3$)

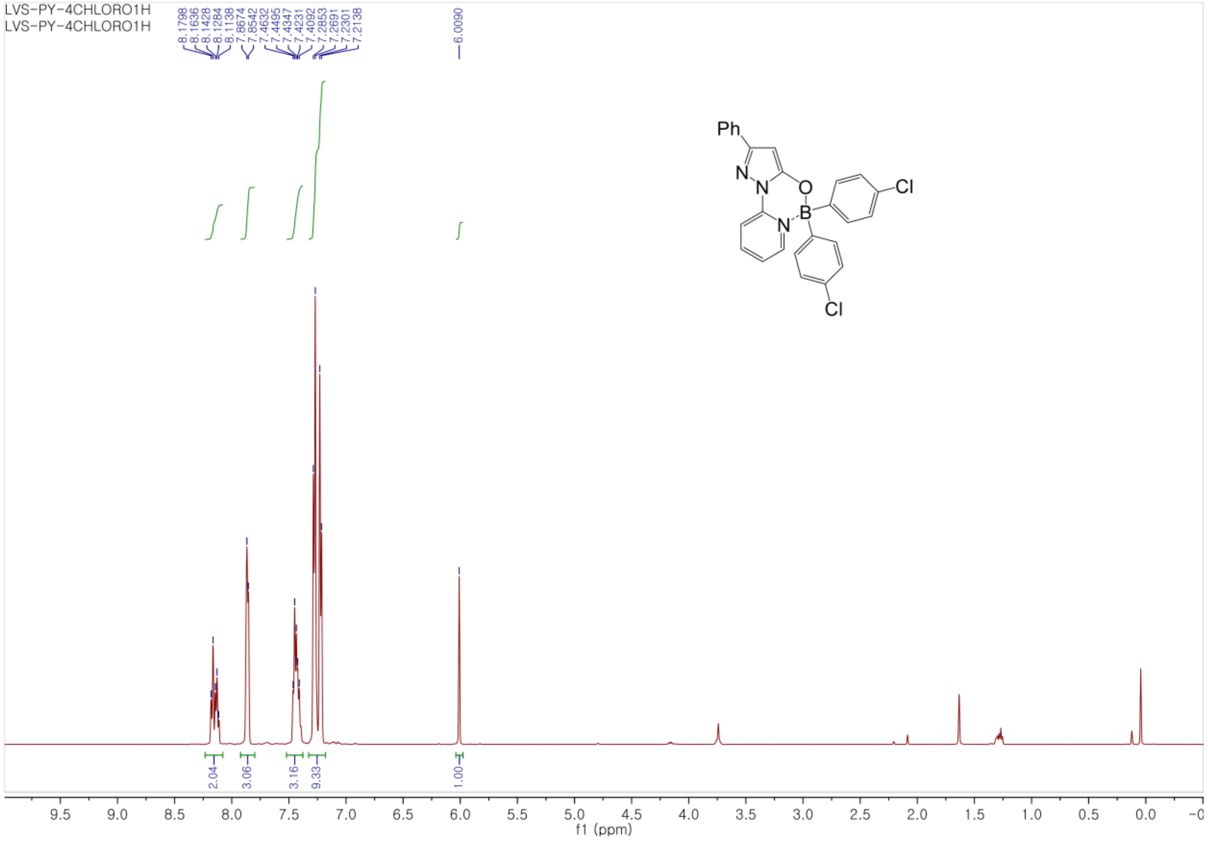

Figure S8. ^{1}H NMR of **2f** (500 MHz, $CDCl_3$)

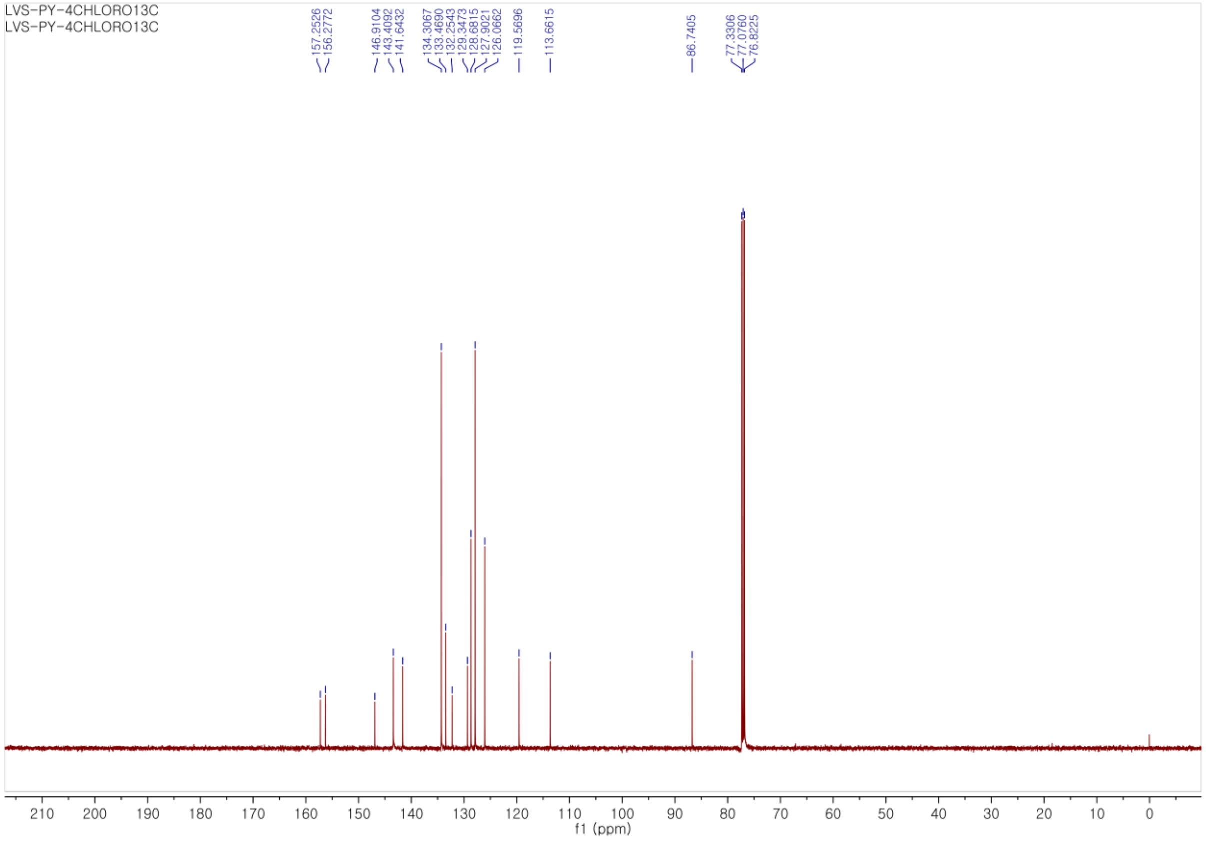

Figure S9. ^{13}C NMR of **2f** (125 MHz, $CDCl_3$)

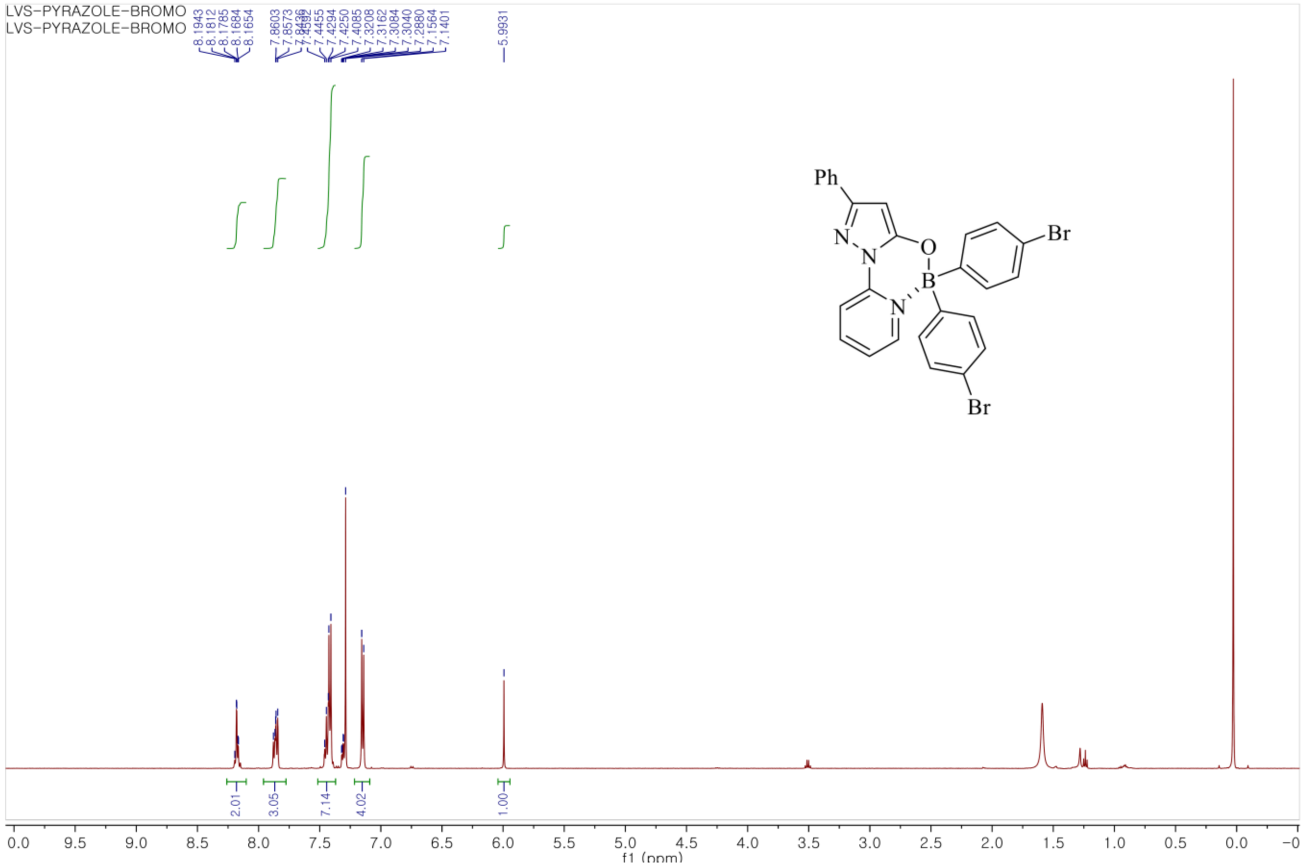

Figure S10. ^{1}H NMR of **2g** (500 MHz, $CDCl_3$)

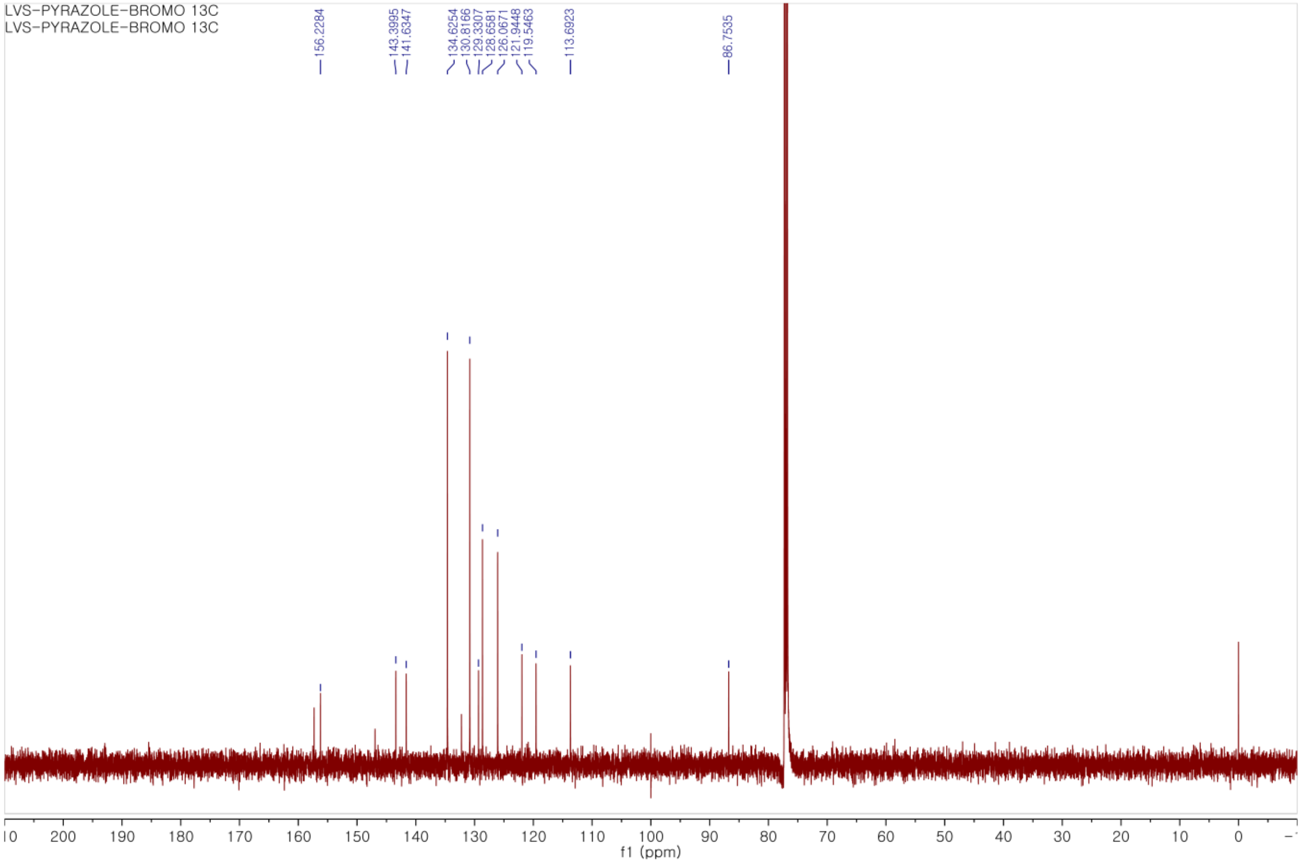

Figure S11. ^{13}C NMR of **2g** (125 MHz, $CDCl_3$)

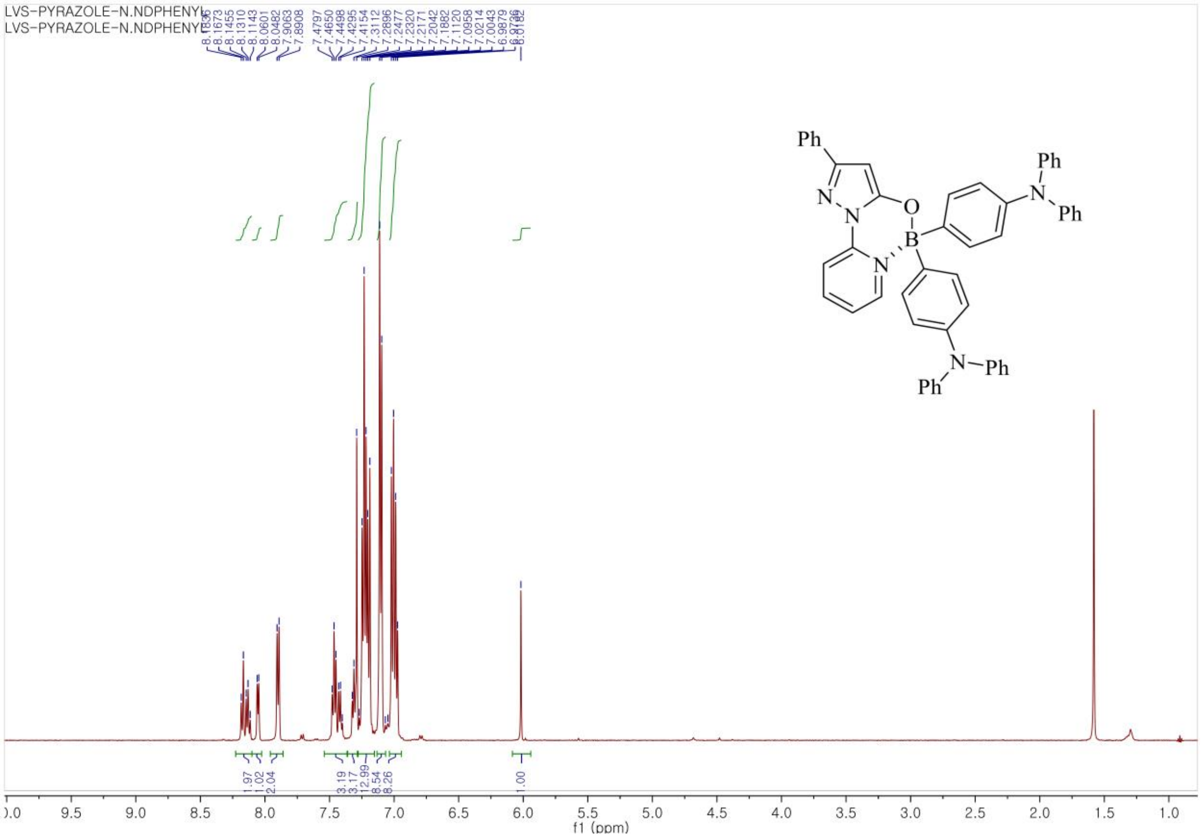

Figure S12. ^{1}H NMR of **2h** (500 MHz, $CDCl_3$)

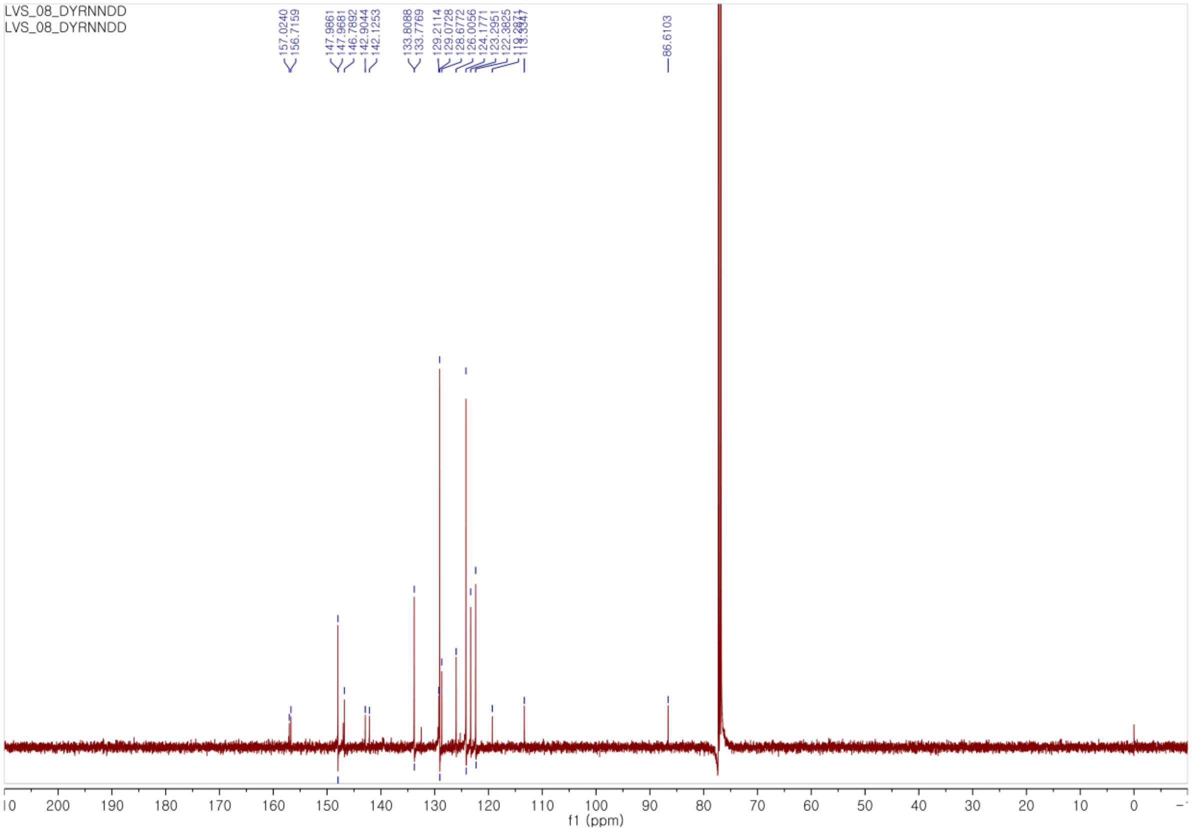

Figure S13. ^{13}C NMR of **2h** (125 MHz, $CDCl_3$)

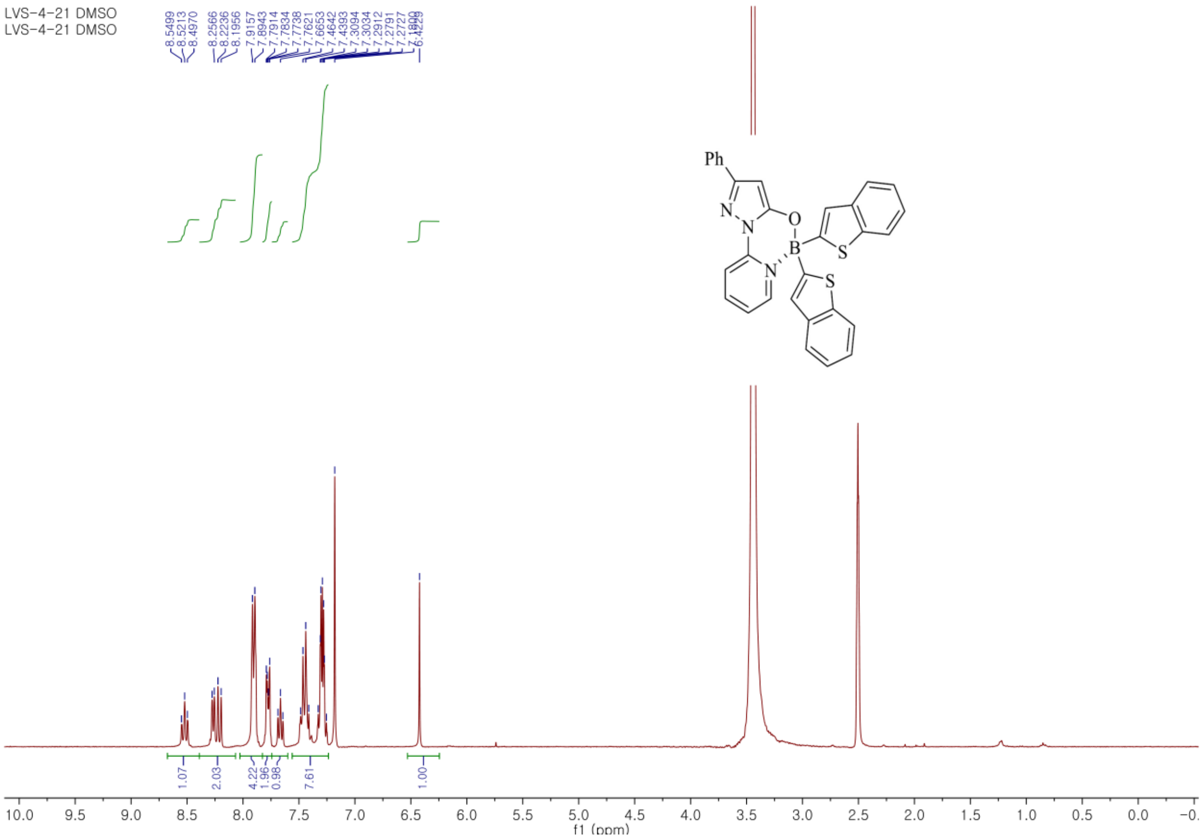

Figure S14. ^{1}H NMR of **2i** (500 MHz, $CDCl_3$)

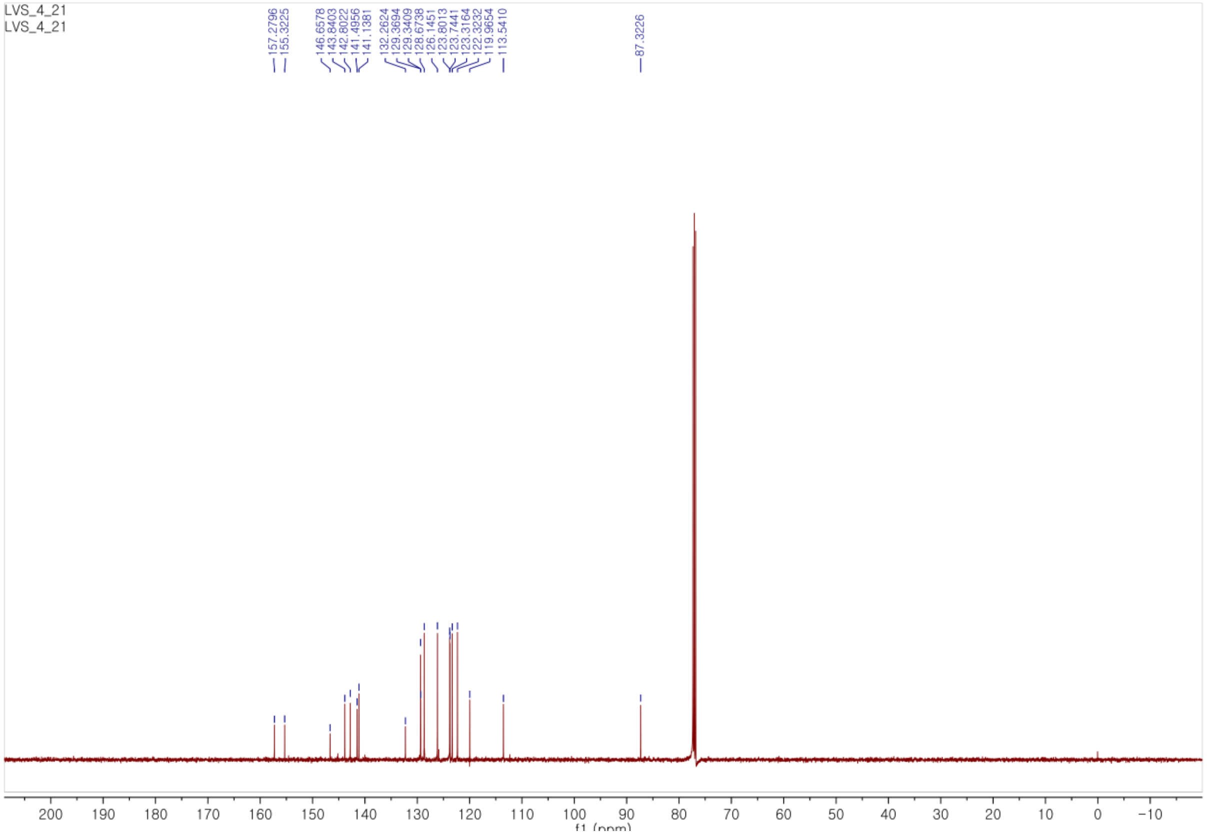

Figure S15. ^{13}C NMR of **2i** (125 MHz, $CDCl_3$)

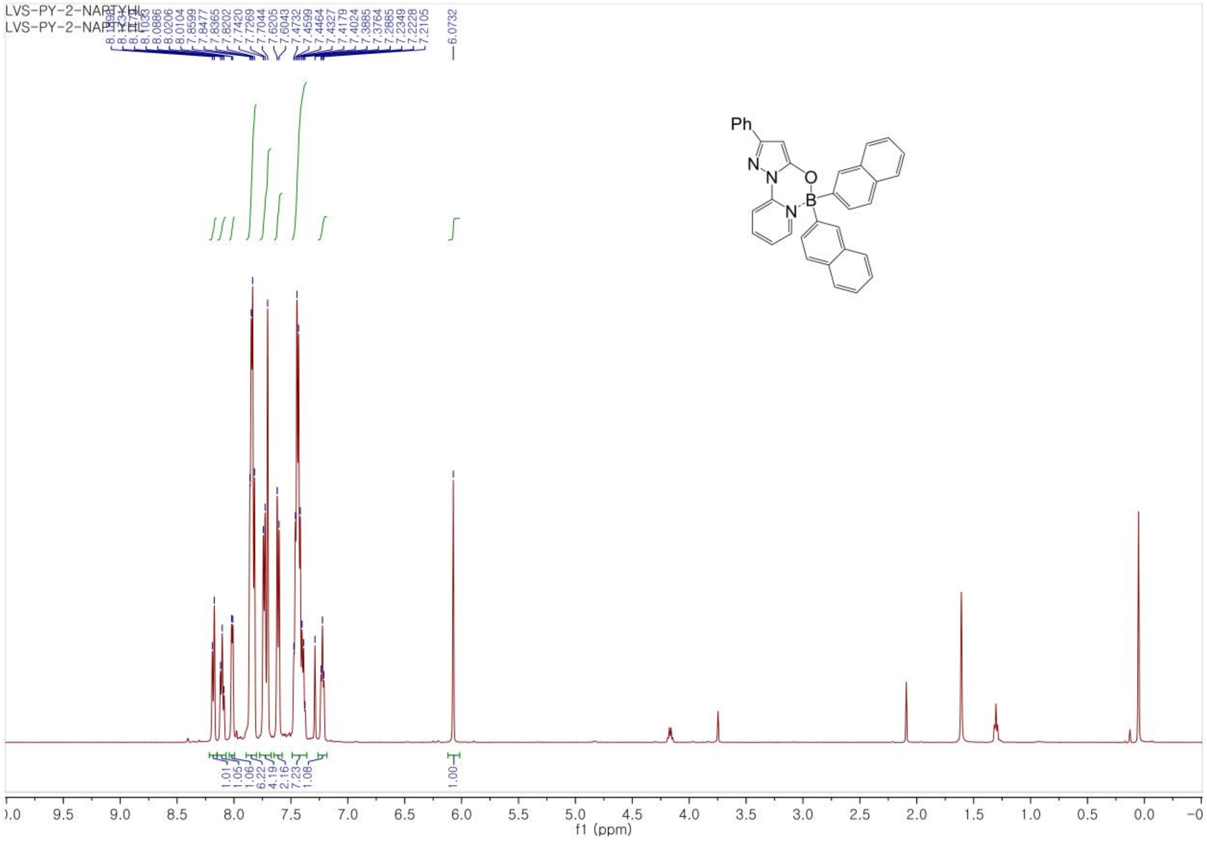

Figure S16. ^{1}H NMR of **2j** (500 MHz, $CDCl_3$)

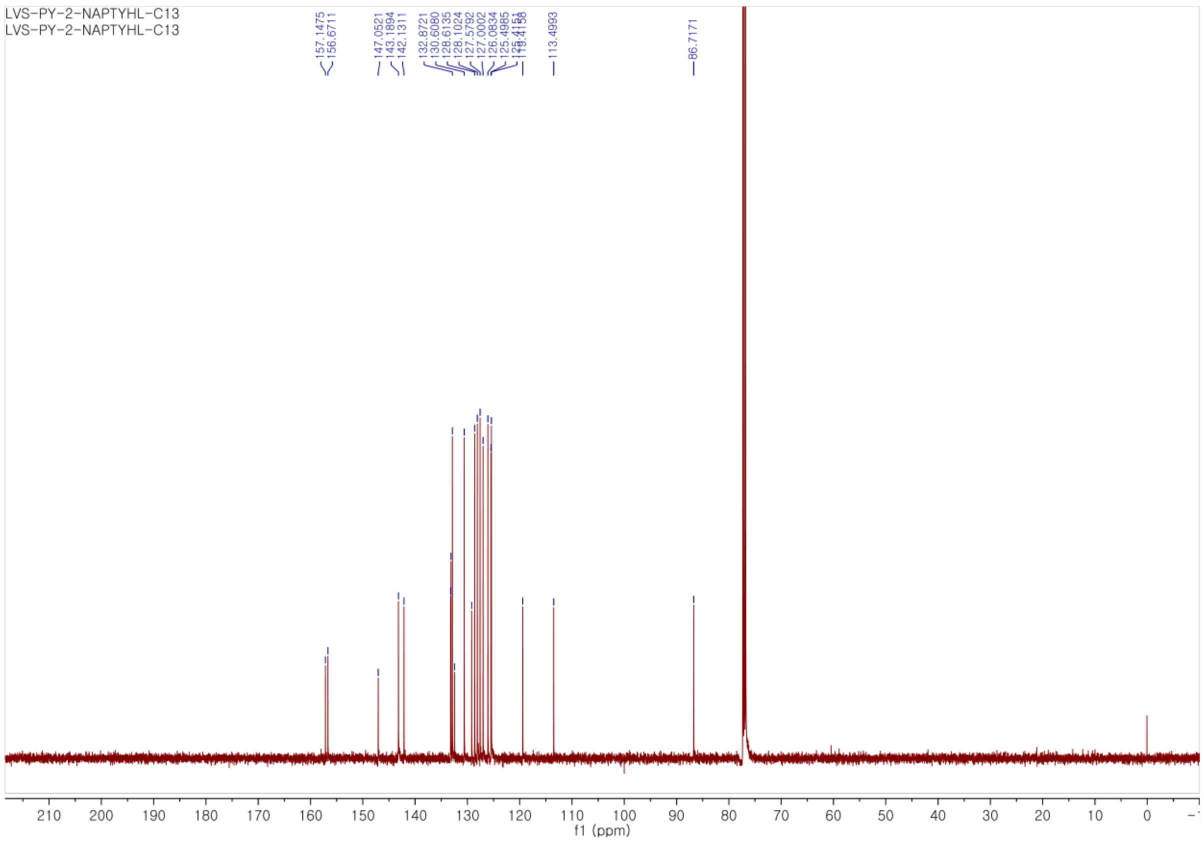

Figure S17. ^{13}C NMR of **2j** (125 MHz, $CDCl_3$)

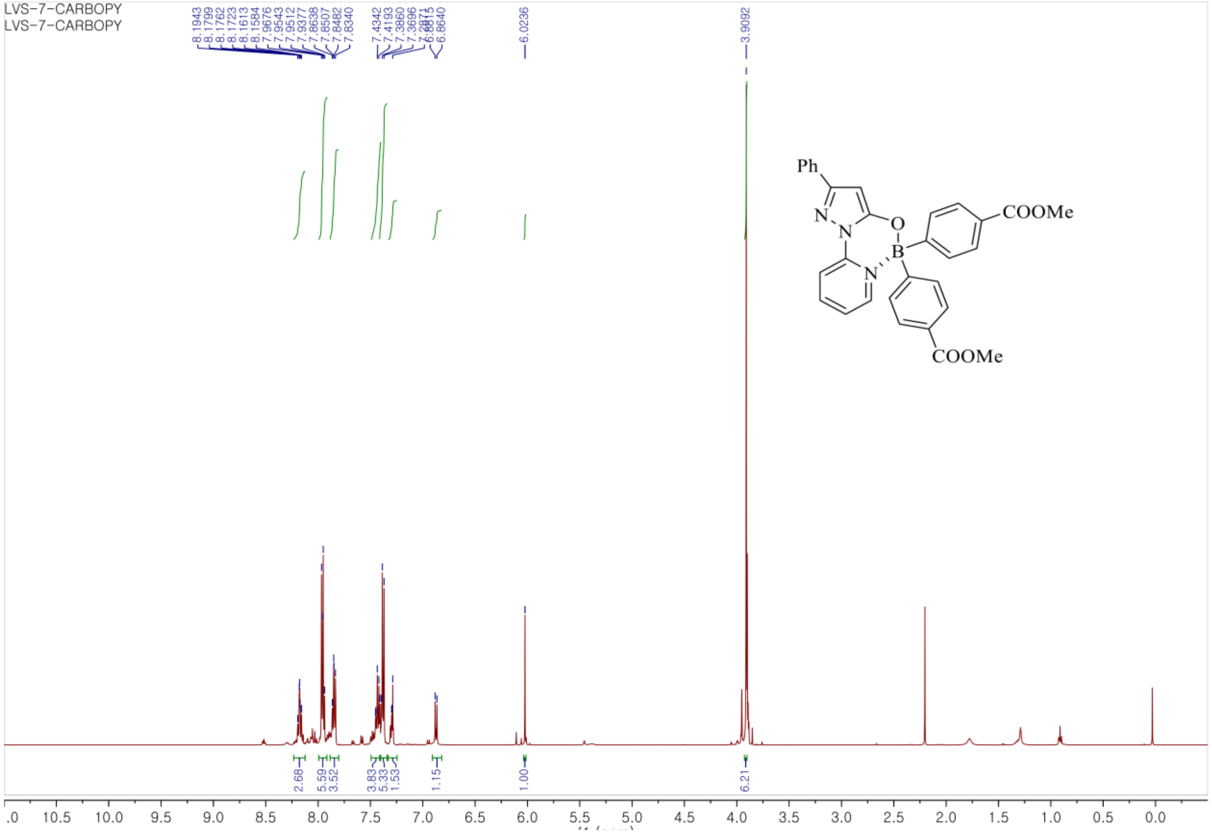

Figure S18. ^{1}H NMR of **2k** (500 MHz, $CDCl_3$)

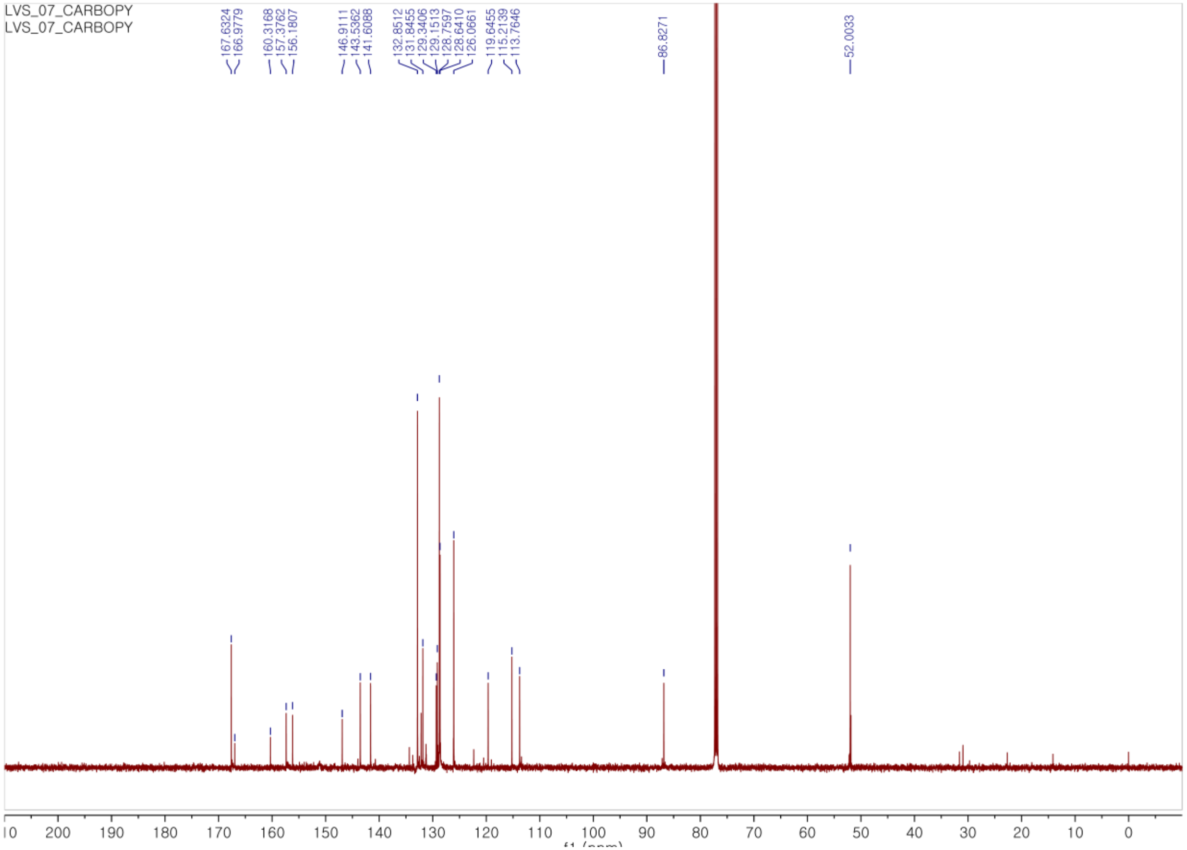

Figure S19. ^{13}C NMR of **2k** (125 MHz, $CDCl_3$)

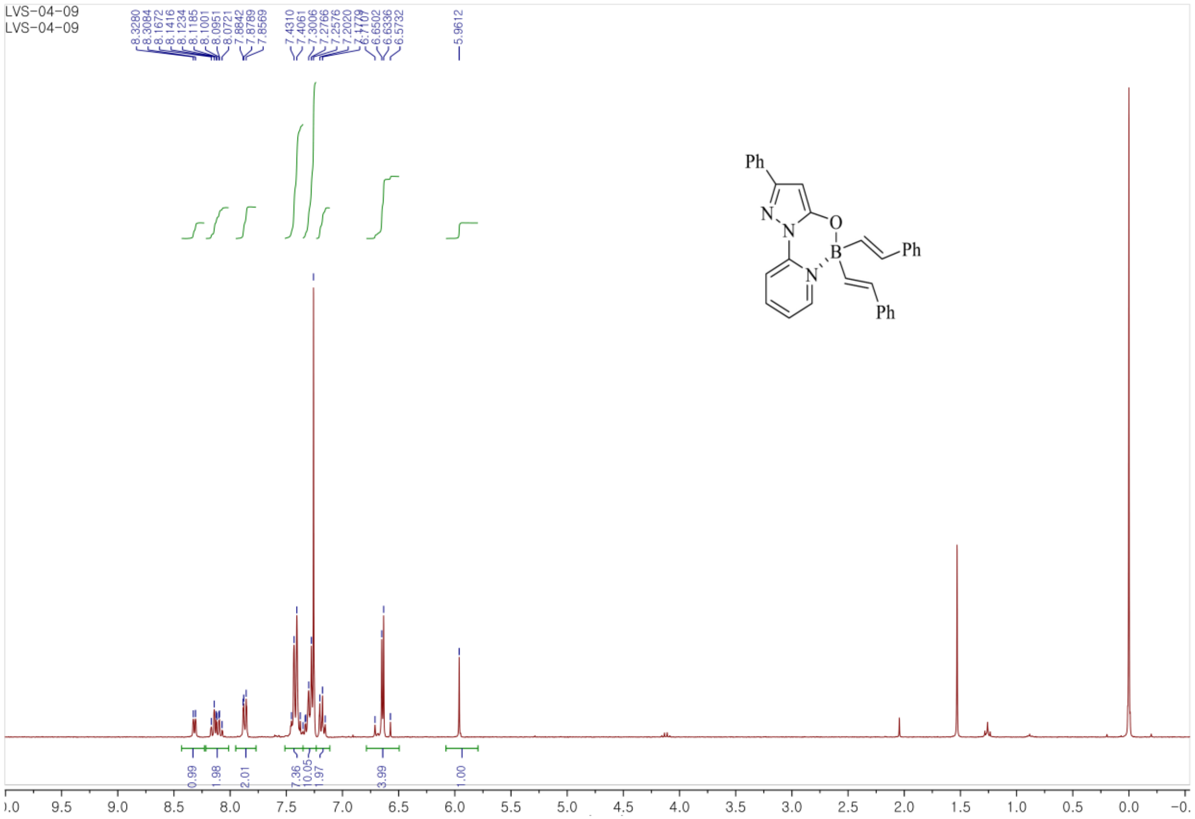

Figure S20. ^{1}H NMR of **2l** (500 MHz, $CDCl_3$)

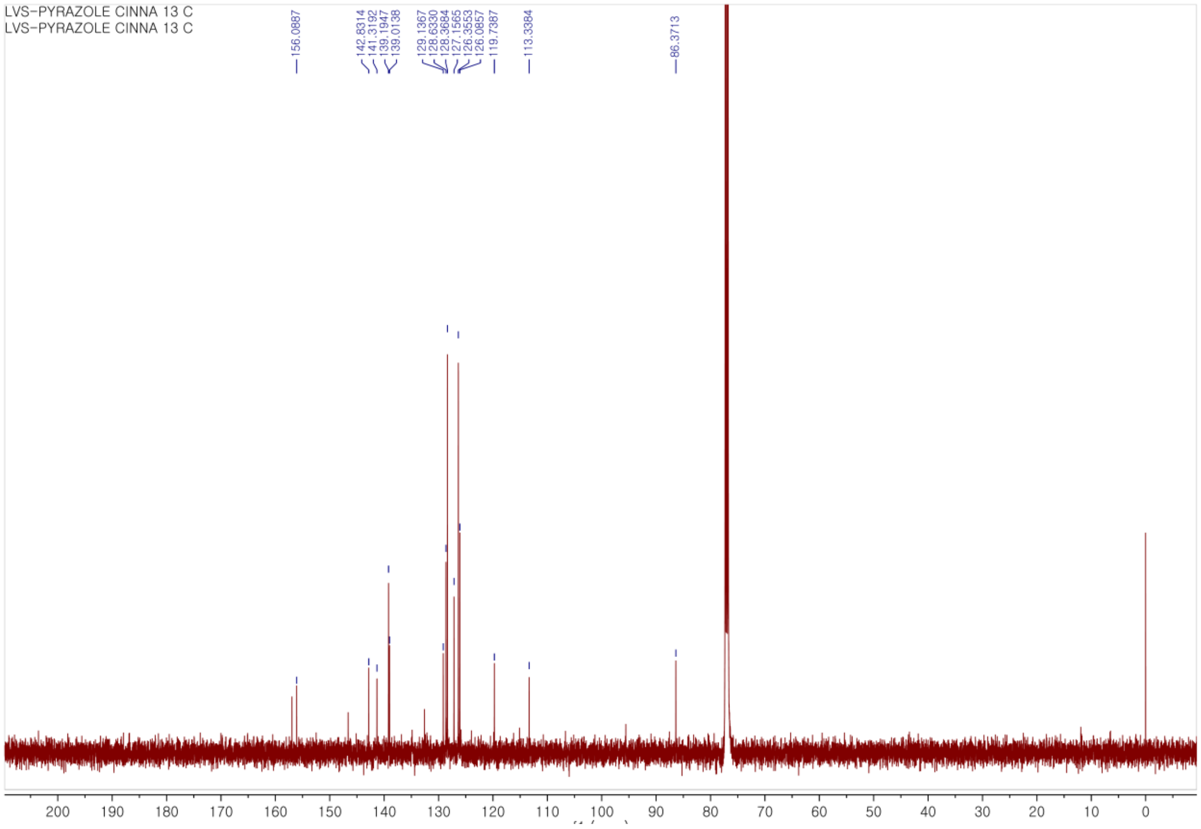

Figure S21. ^{13}C NMR of **2l** (125 MHz, $CDCl_3$)

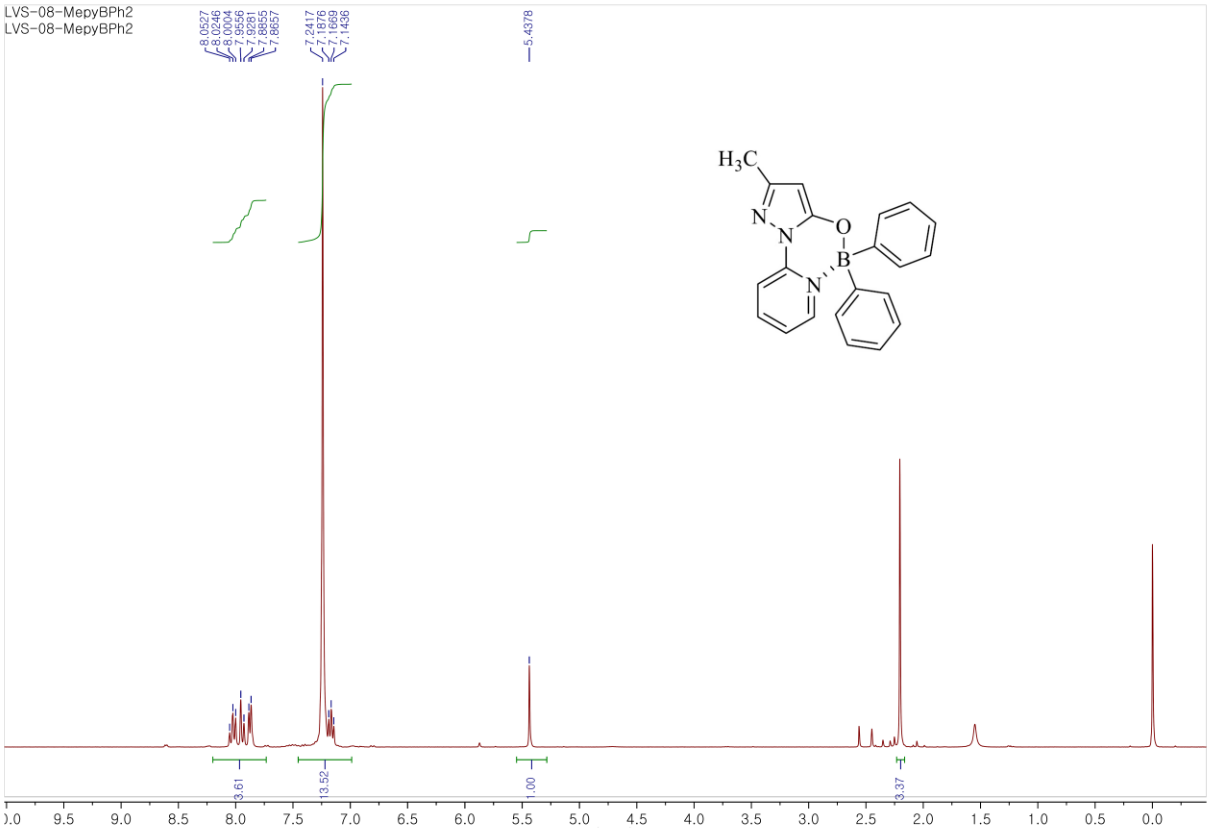

Figure S22. ^{1}H NMR of **2m** (500 MHz, $CDCl_3$)

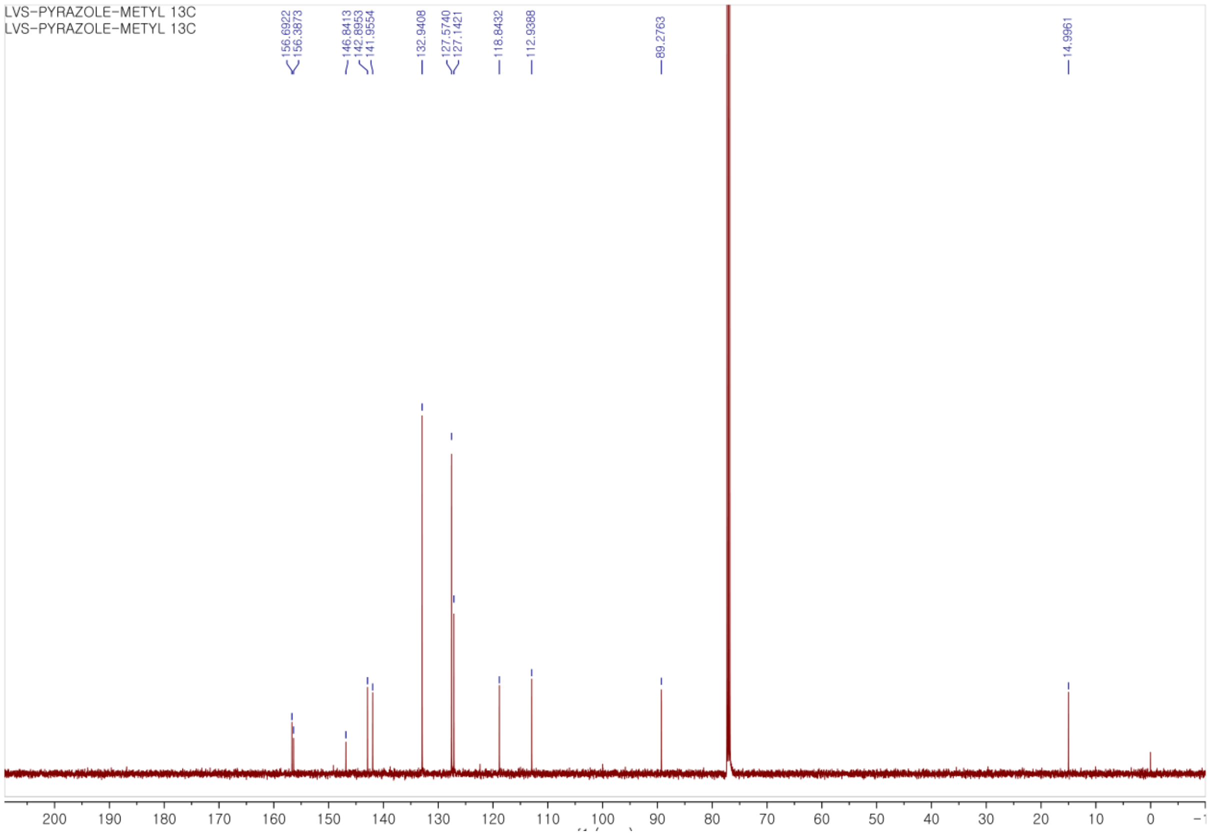

Figure S23. ^{13}C NMR of **2m** (125 MHz, $CDCl_3$)

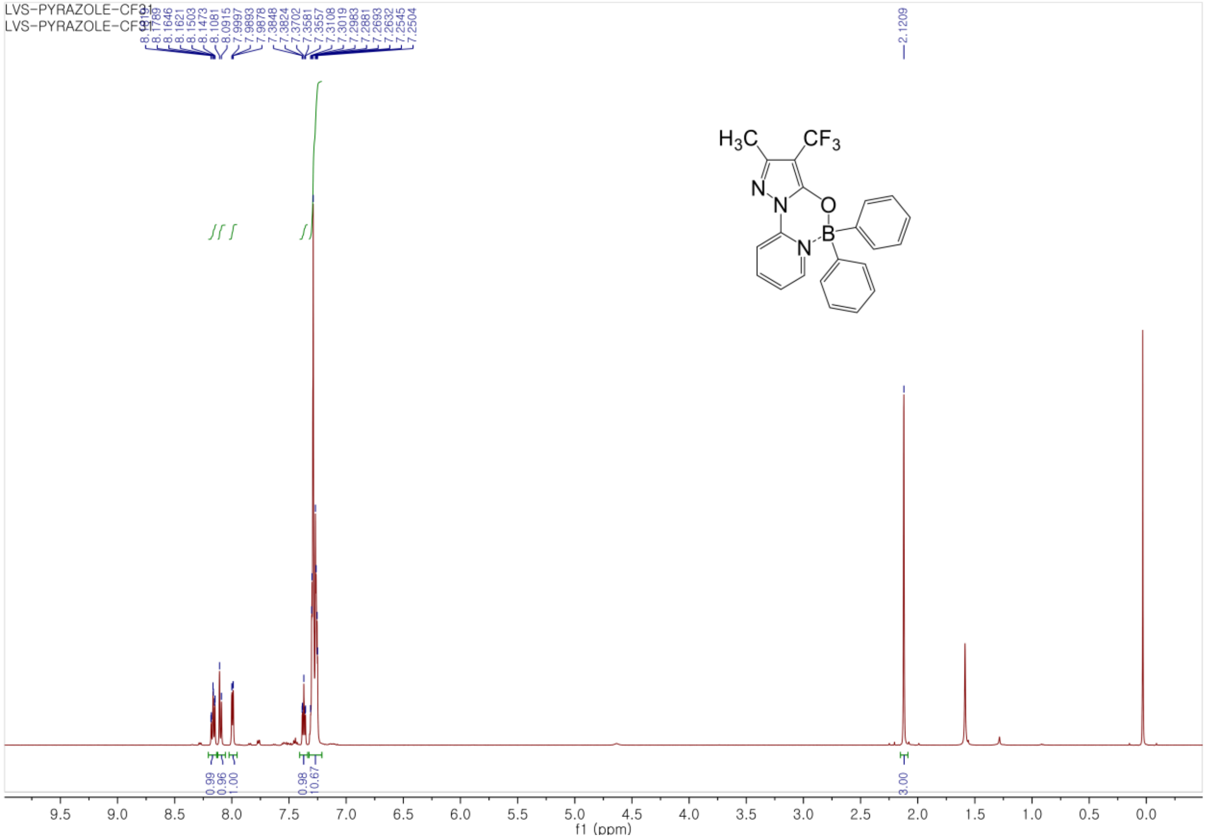

Figure S24. ^{1}H NMR of **2n** (500 MHz, $CDCl_3$)

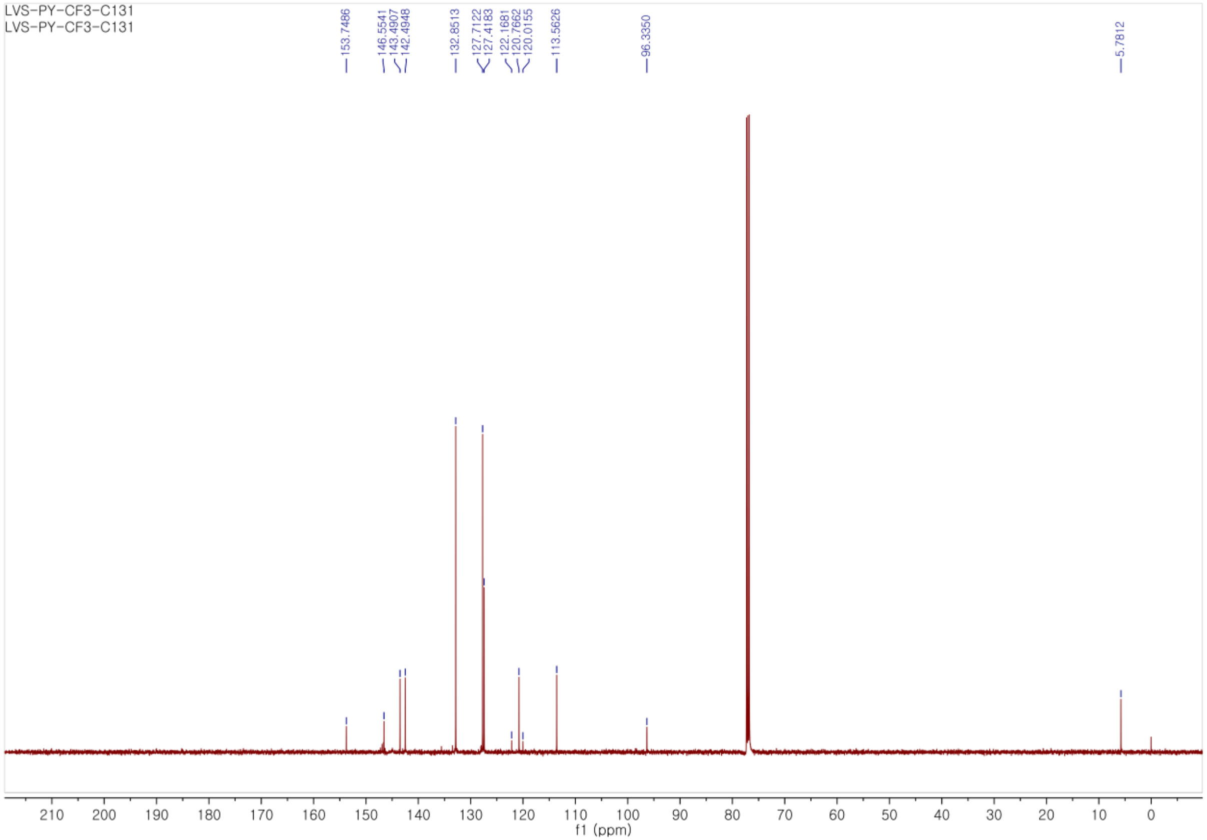

Figure S25. ^{13}C NMR of **2n** (125 MHz, $CDCl_3$)
